# Comparative analysis of genome-wide transcriptional responses to continuous heat stress in *Pleurotus tuoliensis*

**DOI:** 10.1186/s13568-023-01630-y

**Published:** 2023-11-02

**Authors:** Long Chen, Ying Luo, Jiazheng Li, Zhijun Zhang, Di Wu

**Affiliations:** 1 Tianjin Academy of Agricultural Sciences, Tianjin, 300192 China; 2 National Engineering Technology Research Center for Preservation of Agricultural Products, Tianjin, 300384 China; 3 Bionano Genomics, San Diego, CA 92121 USA

**Keywords:** Heat stress response, *Pleurotus tuoliensis*, DEG analysis, Gene function enrichment, Fungal ergosterol biosynthesis

## Abstract

Temperature plays an impactful role in mushroom cultivation. To obtain insights of transcriptomic response in macrofungi against heat stress*,* we performed RNA-seq analysis using *Pleurotus tuoliensis* mycelium cells that were treated under 32 °C and 36 °C for consecutive 96 h. By comparing the growth rate data, we found mycelium cells could maintain normal growth rate almost the same as control under 32 °C, yet halted the growths under 36 °C. In total, 2724 differential expressed genes were identified from the three pair-wise comparisons, which were classified to four clusters based on their expression patterns. We also performed gene set enrichment analysis using both GO and KEGG databases, and revealed 48, 113 and 105 enriched GO terms, and 1, 5, and 6 enriched KEGG pathways for three pair-wise comparisons accordingly. In addition, we identified 9 overlapping GO terms and 1 overlapping KEGG pathway shared by the three comparisons. Differentially expressed genes (DEGs) involved in cell communication, amino acid metabolic process, intracellular signal transduction and small molecule biosynthesis were identified in two heat stress treatments despite of the stress intensity. However, the expression of two heat shock protein genes (*HSP10* and *HSP60*) were induced by increasing temperature. Our findings also suggested the DEGs associated with cell cycle regulation had various expression patterns under two heat stress conditions possibly due to different functions. Furthermore, 11 DEGs related to ergosterol biosynthesis were identified with similar expression trends, indicating the ergosterol levels and cell membrane composition may have a tight connection to the acquisition of thermotolerance, which warrant further investigations for deeper understanding of molecular mechanisms in fungal stress responses.

**Supplementary Information:**

The online version contains supplementary material available at 10.1186/s13568-023-01630-y.

## Key points

- Almost 82% DEGs showed consistent gene regulation trends in both heat stress treatments.
- The study revealed that 9 overlapping GO terms and 1 overlapping KEGG pathway were found across three comparisons, implying several essential biological processes, pathways and organelles involved in mycelial HS response.
- The differential expression levels of ergosterol biosynthesis genes suggests that the ergosterol level may have a significant impact on mycelial acquired thermotolerance.

**Supplementary Information:**

The online version contains supplementary material available at 10.1186/s13568-023-01630-y.

## Introduction

Optimal temperature is crucial for edible macrofungi (mushrooms) as it promotes mycelial growth and nutrition accumulation during the vegetative stage, which prepares the mycelium for fruiting body development (Luo et al. 2021). However, heat stress (HS) can slow down the growth rate, disrupt cell structures and metabolism, impair fruiting body development, and even trigger apoptotic-like cell death of the mycelium (Miles and Chang 2004; Sánchez 2010; Song et al. 2014; Qiu et al. 2018). Mushroom cultivation is always challenged by HS, which costs considerable energy and capital invests to maintain a desirable cultivating temperature (Chen et al. 2022). *Pleurotus tuoliensis* is a novel oyster mushroom species with a crisp texture and high pharmaceutical value (Kong et al. 2012; Zhao et al. 2016; Zhang et al. 2018). However, its production rate is limited by hot weather, as it is highly sensitive to high temperature (Kong et al. 2012; Sakamoto 2018). HS above 35 °C can cause the “spawn-burning” symptom that completely inhibits *P. tuoliensis* mycelium growth, and increases its susceptibility to pathogens (Zhang et al. 2016a). Therefore, understanding how mushrooms respond to HS is vital for breeding of thermally tolerant species and reducing the HS-induced damages.

Recent studies have focused on HS response in mushrooms (Jepsen and Jensen 2004; Mattoon et al. 2021). The heat-shock proteins (HSPs) has significant functions in coping with stress by preventing protein aggregation, removing denatured proteins, and repairing damaged protein folding (Tiwari et al. 2015; Luo et al. 2021; Mattoon et al. 2021). A combined proteome and transcriptome analysis revealed Hsp40, Hsp70, and Hsp90 were involved in the thermotolerance of *Lentinula edodes* (Wang et al. 2018b; Luo et al. 2021; Mattoon et al. 2021). Wang et al. (2018a, 2020) created *LeDnaJ* (*Hsp40*) silenced and over-expressed mutants to characterize the function of *LeDnaJ* during HS response. Their work demonstrated that *LeDnaJ* was essential for mycelial HS resistance.

Trehalose is one of the most widely-studied protective substances in mushrooms (Luo et al. 2021; Mattoon et al. 2021). A comparative analysis of mycelium growth and intracellular trehalose accumulation under HS was conducted between two *Pleurotus pulmonarius* strains, which demonstrated the activities of trehalose metabolic enzymes had remarkable effects on alleviating oxidative damages of cell membranes (Liu et al. 2019). Similarly, Lei et al. (2019) showed that overexpressing the trehalose-6-phosphate synthase gene (*TPS*) in *Pleurotus ostreatus* mycelia lowered the cellular malondialdehyde (MDA) level and increased the capacity to scavenge reactive oxygen species (ROS). Additionally, several signaling molecules, such as Ca^2+^, H_2_S, and NO, were identified and demonstrated with specific functions in HS signaling transduction. These molecules facilitated mushrooms to adjust their physiology and genetics to mitigate the cellular damages caused by HS (Kong et al. 2012; Zhang et al. 2016b; Tian et al. 2019; Hou et al. 2020a).

Previous investigations have explored how certain metabolic pathways, proteins (genes), protective substances, and signaling molecules are related to HS response. These studies have contributed valuable findings to understand how fungi cope with HS at the molecular level. However, eukaryotic HS response is a complex biological process that involves numerous biochemical reactions, metabolic alterations, and physiological adjustments. These processes are regulated by genetic and/or epigenetic regulations that are not fully understood (Liu et al. 2015). Therefore, more comprehensive and integrative studies are needed to reveal the mechanisms of fungal HS response at different levels.

Multi-omics approaches enable researchers to perform comprehensive analyses to obtain the insights of molecular mechanisms behind the fungal HS response on a broader scope (Wang et al. 2017, 2018c; Hou et al. 2020b; Yan et al. 2020). For instance, Tan et al. (2018) used RNA-Seq to identify 176 differential expressed genes (DEGs) related to carbohydrate metabolism and other pathways under HS at 42 °C for 2 h in *Ganoderma lucidum* mycelia. Zou et al. (2018) used iTRAQ to detect proteomic changes in *P. ostreatus* mycelia under HS (40 °C for 48 h) and recovery phase. Their results indicated proteins involved in MAPK signaling, antioxidant defense, HSPs production, and glycolysis pathway were critical for thermotolerance enhancement. A comparative proteomics analysis by Xu et al. (2021) in *Hypsizygus marmoreus* revealed enzymes related to catalases, superoxide dismutases, peroxidases, as well as trehalose synthesis enzymes were up-regulated under HS (37 °C for 8 h and 24 h). However, these studies are limited by the lack of reference genome and gene function annotations of some species (Zhang et al. 2018). Thus, more integrative studies are needed to elucidate the genetic regulations of fungal HS response.

We performed RNA-Seq to analyze gene expression alterations of *P. tuoliensis* mycelia under HS at 32 °C, and 36 °C for 96 h, and observed divergent mycelial growth rates under different HS intensities. Key genes and pathways participating the HS response were identified through comparative analyses of physiological and transcriptomic data. Our study aimed to provide a deeper insight into the genome-wide fungal HS response at the transcription level in *P. tuoliensis*.

## Materials and methods

### Fungal strains and growth conditions

The *P. tuoliensis* strain used in this study is Zhongnong No.1 (CCMSSC00489) provided by the China Center for Mushroom Spawn Standards and Control (CCMSSC) (Wang et al. 2018d, 2022). The mycelia of *P. tuoliensis* were cultured on potato dextrose agar (PDA; BD Difco, Franklin Lakes, USA) plates, which were divided into three groups: control (CK), moderate heat stress treatment (MHT), and severe heat stress treatment (SHT). To ensure all mycelia had equally initial viability, the inoculum in each treatment was prepared from the same initial plate (full of mycelium) with a $\varnothing$ 3 mm cutter. All the plates were incubated in the dark at 25 °C for 9 days after inoculation.

### Continuous HS treatments and mycelia colony area quantification

On the 10th day after inoculation, plates in the MHT and SHT groups were transferred to incubators set at 32 °C and 36 °C, respectively, while plates in the CK group remained at 25 °C. After 96 h of continuous HS treatment, mycelia from three individual plates in each group were scraped and immediately frozen in liquid nitrogen for further analysis.

To quantify mycelial colony area, we measured the colony diameter, and developed an algorithm based on high-resolution plates images. Images were taken on the 9th and 13th days with respect to before and after HS. Each image was converted to black and white, and was re-sized to a constant size for subsequent processing. The algorithm read the red category of red, green and blue (RGB) values for each pixel, and calculated their frequency. Two critical thresholding RGB values were selected manually to screen pixels for Petri dish and mycelial colony domains. As Eq. (1) shows, the number of pixels in a dish area as index ${A}_{d}^{i}$ and the number of pixels in a colony area as index ${A}_{c}^{i}$. The ratio (*η*) between these indexes was named as the normalized colony area ratio (NCAR), indicating the approximate percentage of colony coverage on the Petri dish. The NCAR mean value was calculated for each group based on the number of replicate samples (*n*). The documentation and source codes of the NCAR algorithm were deposited on GitHub (Chen 2022).

1 $$\eta =\frac{\sum_{i}^{n}\left({A}_{c}^{i}/{A}_{d}^{i}\right)}{n}\times 100\%\,\, i \in [1, \dots , n]$$

### RNA extraction and RNA-Seq

The total RNA was extracted and purified from individual frozen mycelia sample using the HiPure Universal RNA Mini kit (Magen, Guangzhou, China). After checking the quality and concentration of the total RNA samples, we constructed cDNA libraries following the Illumina RNA-Seq protocol. The sequencing was conducted on an Illumina NovaSeq 6000 platform with a 150 bp paired-end reading length (PE150) at Magigene Biotechnology Co. Ltd. (Guangzhou, China). The RNA-Seq data can be accessed in the NCBI database under the project accession number PRJNA889562.

### Transcriptome assembly and annotation

Quality control and preprocessing of raw reads were conducted using the fastp software (Chen et al. 2018) to filter out low-quality reads and ensure the individual lengths must be above 75 bp. In addition, to minimize the interference of rRNA, preprocessed raw reads were further trimmed by removing aligned rRNA sequences via Bowtie2 (Langmead and Salzberg 2012; Langmead et al. 2019). We mapped the RNA-seq reads against the reference genome of *P. tuoliensis* strain JKBL130LB (Kim et al. 2019)) using HISAT2 to identify exon-exon splice junctions (Kim et al. 2019). We assembled aligned RNA-Seq reads into potential transcripts using StringTie (Pertea et al. 2015). Unigenes were attributed to the transcripts matching the protein encoding sequences of the reference genome, which were subsequently annotated using BLASTX and the Non-Redundant (NR) protein database (Boratyn et al. 2019).

### Identification of DEGs and expression patterns

The data of raw counts were further cleaned up by removing those unigenes that had zero counts in any samples via R software (Andy Bunn 2017). The raw counts were then normalized using the package DESeq2 version 1.34.0 (Love et al. 2014) to compare the gene expression levels between CK, MHT, and SHT quantitatively. DEGs were identified according to statistical significance analyses using the criteria of adjusted *p* value < 0.05 and absolute log_2_ fold-change > 1 in any pair-wise comparison (CK & MHT or MHT & SHT or CK & SHT). We classified all identified DEGs into distinct clusters based on their expression patterns across three groups of treatments using a divisive hierarchical clustering algorithm with DEGreport version 1.32.0 (Pantano 2022).

### Functional enrichment analysis

The functional enrichment analysis was performed using Gene Set Enrichment Analysis (GSEA) algorithm embedded in the package clusterProfiler version 4.2.2 (Subramanian et al. 2005; Wu et al. 2021), including both Gene Ontology (GO) annotation and KEGG pathways analyses(Kanehisa and Goto 2000; Ashburner et al. 2000; Kanehisa 2019; Carbon et al. 2021; Kanehisa et al. 2021). Due to the limited annotation references for *Pleurotus* genus, we annotated genes functions using the reference genome annotation data of *Saccharomyces cerevisiae* (Carlson 2019).

## Results

### Mycelial growth rate characterization under continuous HS treatment

The images of colonies indicated that the three groups had identical initial sizes on Day 9, but the CK and MHT groups grew much faster compared to the SHT group on Day 13 (Additional file 1: Fig. S1). The CK group had more robust mycelium at the colony edges and a large amount of young mycelium. However, the MHT group had thicker and crustier edges for their colonies.

**Fig. 1:**
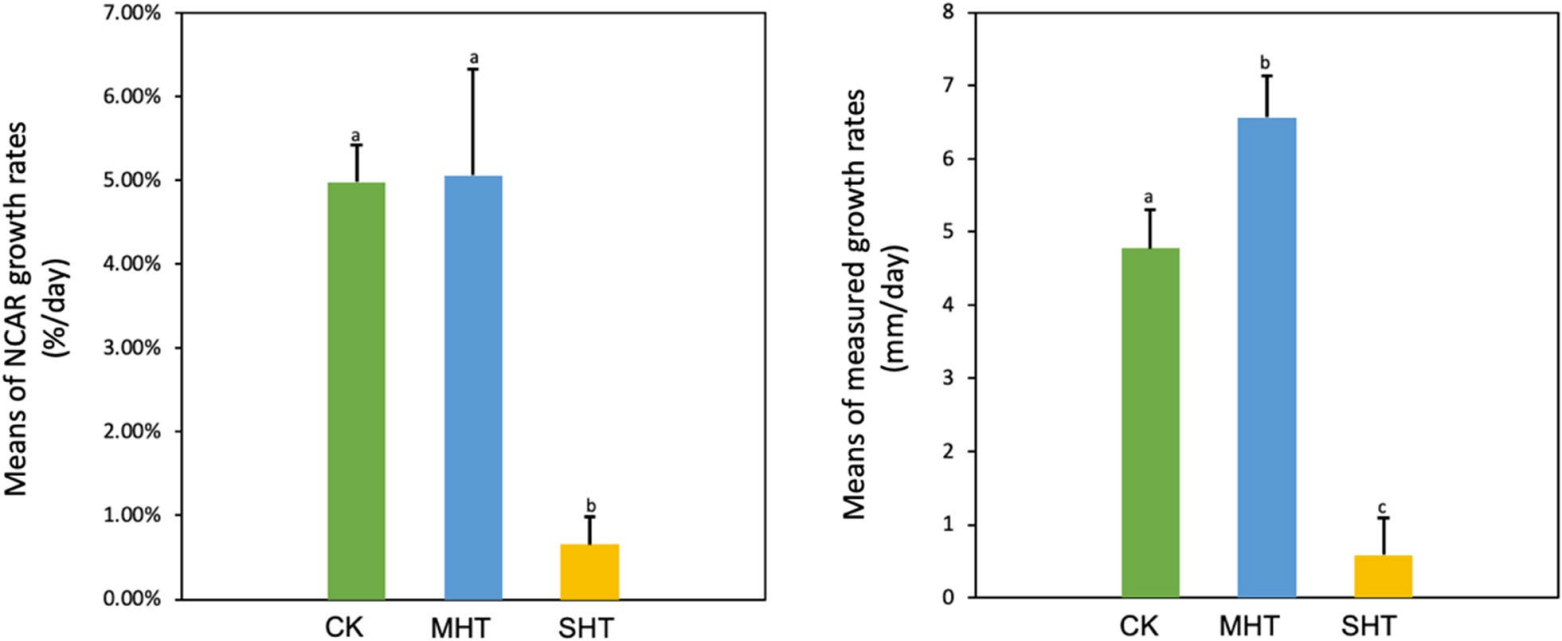
Bar plots of mean mycelial growth rates from Day 9 to Day 13 based on NCAR data and experimental measurements. Groups with different letters are statistically different at 95% family-wise confidence level. Error bars represent standard deviation

Both NCAR and experimentally measured mycelial growth rates implied the effects of different levels of HS on the growth of mycelial growth (Fig. 1). During 96 h of continuous HS treatment, the average NCAR growth rate of the MHT group was 5.06% per day, showing no statistically significant difference compared to CK group. However, the MHT group had a 7.7-fold higher growth rate than the SHT group. The experimental measurements revealed the average growth rates of the CK, MHT and SHT groups were 4.77, 6.56, and 0.58 mm/day respectively, indicating that the MHT group grew even faster than the CK group while the SHT group had the minimal growth rate. Note, the SHT colonies were able to resume growth at a recovery temperature of 25 °C after HS.

### RNA-seq, transcriptome assembly and annotation

We performed initial quality control on the raw reads from each sample and obtained roughly 17 to 26 million clean paired reads with a clean data ratio above 91% and an average GC content of 53.45% (Table 1). The total paired reads were further cleaned up by removing the rRNA sequences (Additional file 1: Table S1). On average, 91.31% of the total clean reads were mapped to the reference genome (*P. tuoliensis* strain JKBL130LB) (Additional file 1: Table S2), generating 26,989 unigenes of which 13,953 unigenes had substantial expression levels. We further removed the unigenes that lacked annotation information in the Nr database via BLASTX (Boratyn et al. 2019), resulting in a final set of 11,265 unigenes for subsequent quantitative analysis.

**Table 1:** Summary statistics of sequencing samples

Sample	Clean paired reads	Clean bases (G)	Q20 (%)	Q30 (%)	GC content (%)	Clean data ratio (%)
CK1	16,904,798	4.94	99.16	96.6	53.74	93.79
CK2	22,009,157	6.4	98.94	95.91	53.49	91.62
CK3	21,485,183	6.23	99.08	96.36	53.48	92.56
MHT1	26,032,345	7.57	98.95	95.89	53.43	91.92
MHT2	23,893,148	6.94	99.03	96.14	53.5	91.95
MHT3	23,676,604	6.87	98.96	95.94	53.18	91.35
SHT1	26,241,571	7.61	99.04	96.18	53.48	91.52
SHT2	23,261,438	6.74	99.16	96.59	53.44	92.86
SHT3	23,375,530	6.79	98.97	95.94	53.32	91.56

CK1, CK2 and CK3: three independent biological replicates of Control

MHT1, MHT2 and MHT3: three independent biological replicates of group MHT

SHT1, SHT2 and SHT3: three independent biological replicates of group SHT

Q20: the percentage of bases with a Phred score over 20

Q30: the percentage of bases with a Phred score over 30

### Expression quantification and DEG identification

**Fig. 2:**
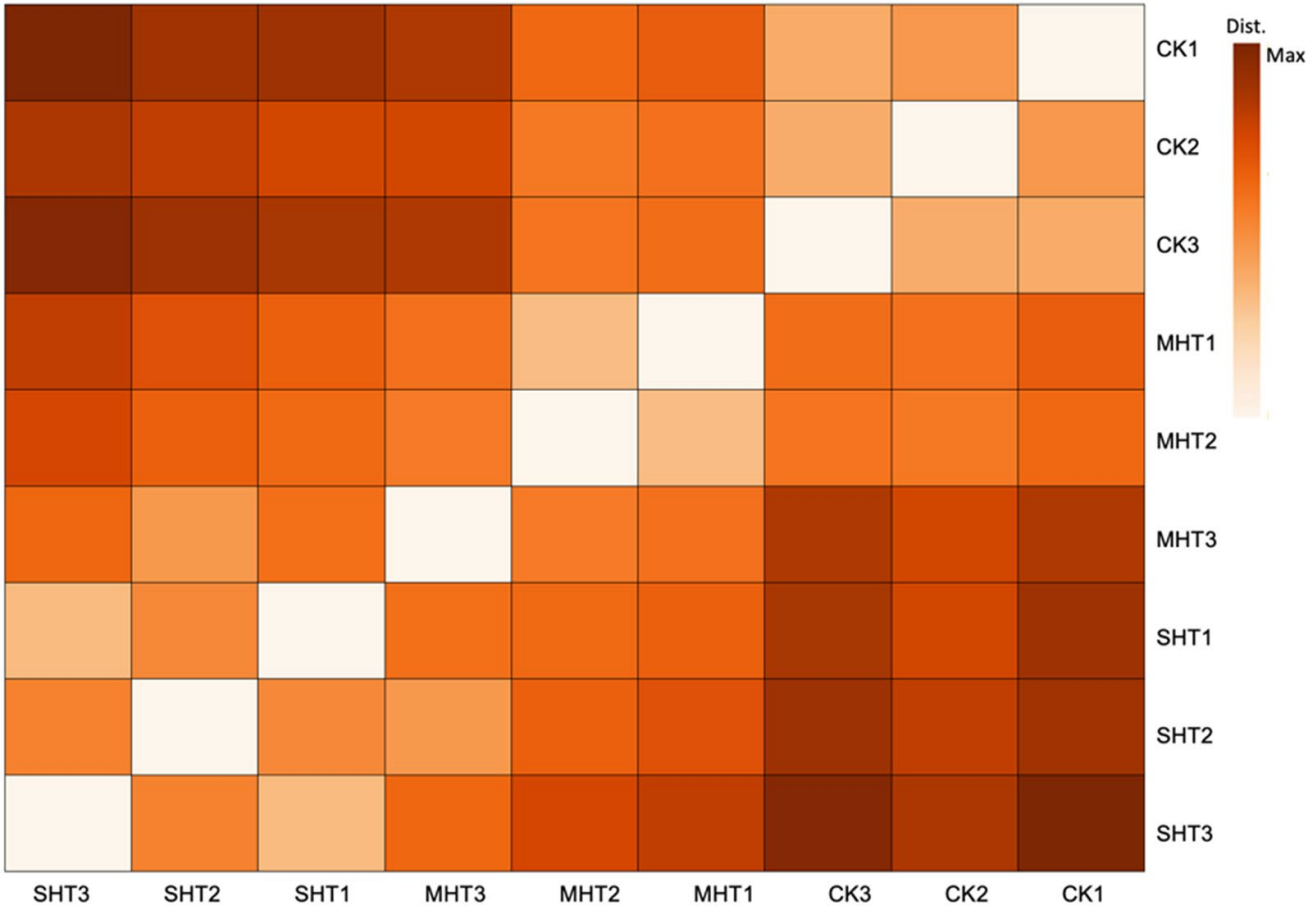
A heatmap depicts orthogonal distances between nine samples based on the normalized counts of the entire 11,265 unigenes. The darkest color indicates the maximum distance between two individual samples

Both consistencies and divergencies were observed across the 9 samples in three groups by comparing the normalized counts of the entire 11,265 unigenes. We checked the variability of all unigenes normalized counts by creating a dispersion plot, which showed the dispersion estimates were shrunk towards a fitted curve (Additional file 1: Fig. S2). In addition, relatively smaller distances were found between the triplicates of the same group as expected, indicating overall consistencies in gene expression under the same treatment (Fig. 2). Whilst the contrasts between the SHT and the CK samples possessed rather darker color, presenting obvious divergencies in overall gene expression. Interestingly, the distances between the MHT and CK groups indicated reduced divergencies compared to those between SHT and CK, which may be related to an intermediate HS treatment.

**Fig. 3:**
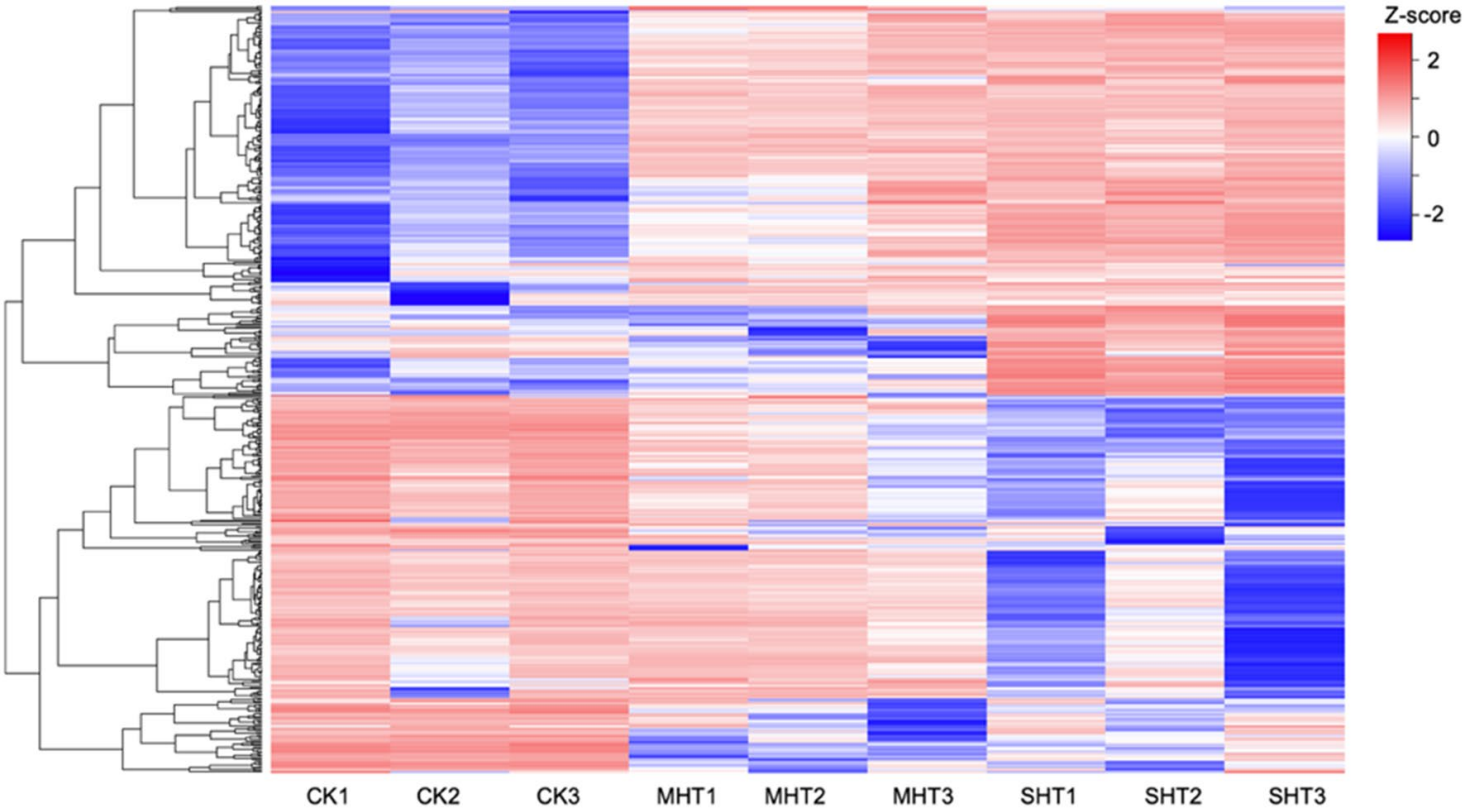
Top 500 unigenes with the largest variations of normalized counts across 9 samples. Each row corresponds to a single unigene and the dendrogram on the left shows the distances between unigenes. The legend shows the gene expression level (Z-scores) calculated based on the normalized counts. Each column represents a specific sample listed at the bottom

Up to 500 unigenes with the maximum variations of normalized counts were selected to visualize the divergency in gene expression across samples. The color changes from left to right in Fig. 3 show clear differences between three groups of treatments, as well as overall consistency within each group. A cluster of unigenes in the upper half showed relatively low expression levels in CK samples, as indicated, the blue bars, but increased expressions in MHT and SHT samples. Conversely, a cluster of unigenes in the bottom half showed high expression level in CK, while reduced expressions in MHT and SHT. A few unigenes in the middle had similar low expression levels in CK and MHT groups marked by blue bars, however high expression levels in SHT group. At the bottom of Fig. 3, a series of unigenes had high expression levels in CK group, although the lowest expression levels in MHT group rather than in SHT group.

**Fig. 4:**
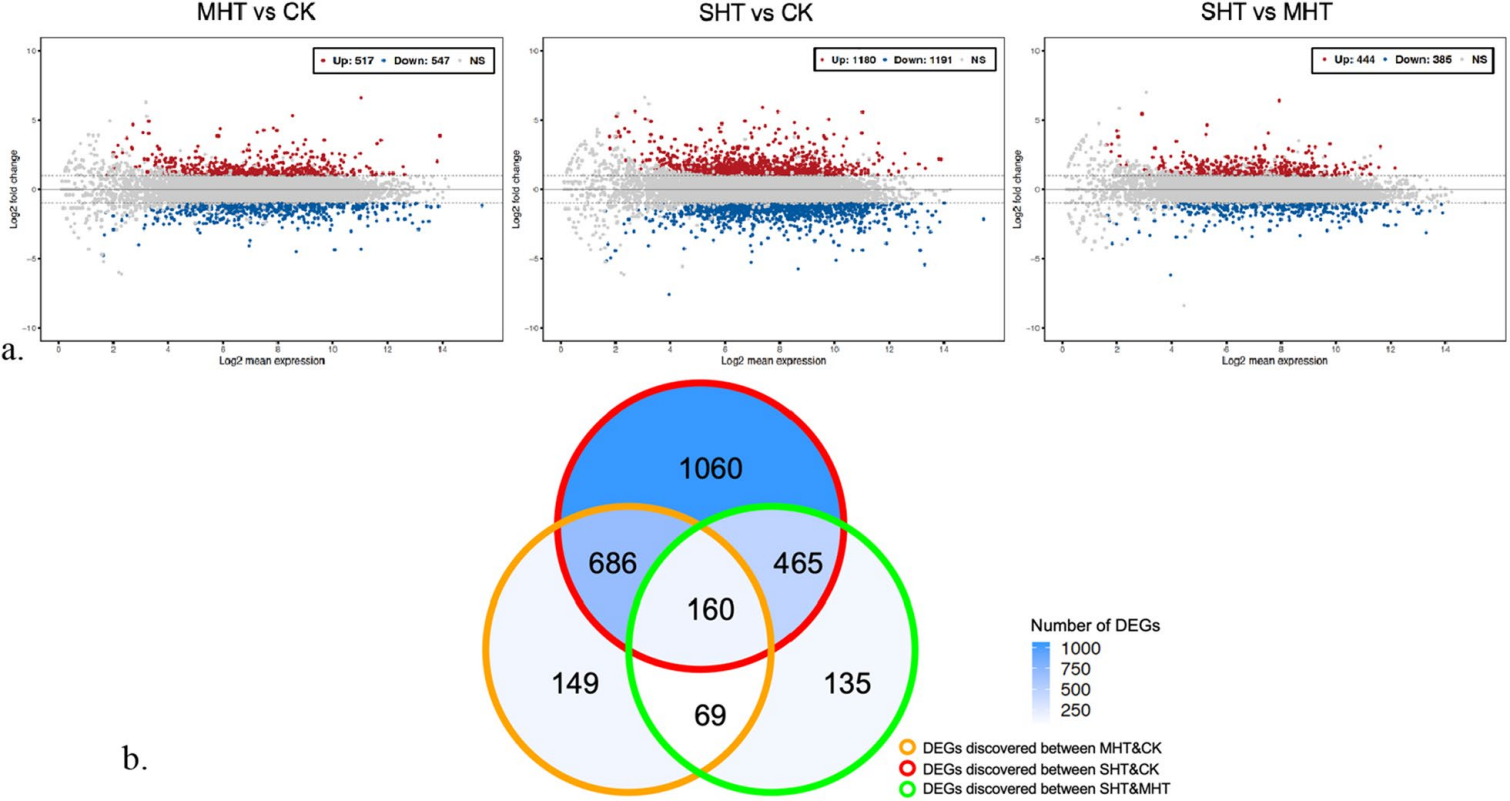
Illustrations of total 2724 DEGs with specifications. **a** MA plots showing the expression levels and fold-changes of the DEGs detected in MHT&CK, SHT&CK, and SHT&MHT. The x-axis is the log_2_ value of normalized counts, and the y-axis stands for the log_2_ fold-change of each unigene. Grey dots (NS) are non-DEGs, red dots (UP) are up-regulated DEGs, and blue dots (DOWN) are down-regulated DEGs; **b** a Venn diagram showing the distribution and overlap of the DEGs in each comparison

According to the quantitative analysis, we detected 2724 DEGs out of 11,265 unigenes that met the screening criteria of |log_2_-fold change| > 1 and *p-adj* value < 0.05 in any of the three comparisons: MHT&CK, SHT&CK, and SHT&MHT. Under HS at 32 °C, 1064 DEGs were detected compared to the CK, while the number of DEGs elevated to 2371 when the HS temperature increased to 36 °C (Fig. 4a). Only 829 DEGs were detected based on the comparison of SHT&MHT. The up-and-down regulations of DEGs varied slightly in each comparison. The number of down-regulated DEGs was 5.8% greater than that of up-regulated in the comparison of MHT&CK, while the DEGs detected between SHT and CK groups indicated an almost equalized number of up-regulations and down-regulations (Fig. 4a). Remarkably, more up-regulated than down-regulated DEGs were found in the comparison of SHT&MHT.

Both unique and overlapped DEGs were found in the three pair-wise comparisons (Fig. 4b). We found that 1060 DEGs were unique to SHT&CK, accounting approximately 39% of the total number of DEGs. Conversely, only 149 and 135 unique DEGs were specific to the comparisons of MHT&CK and SHT&MHT, respectively. The largest overlap of DEGs was between MHT&CK and SHT&CK with 846 common unigenes. Moreover, 160 DEGs were shared by all three comparisons.

### Classification of DEG expression patterns

**Fig. 5:**
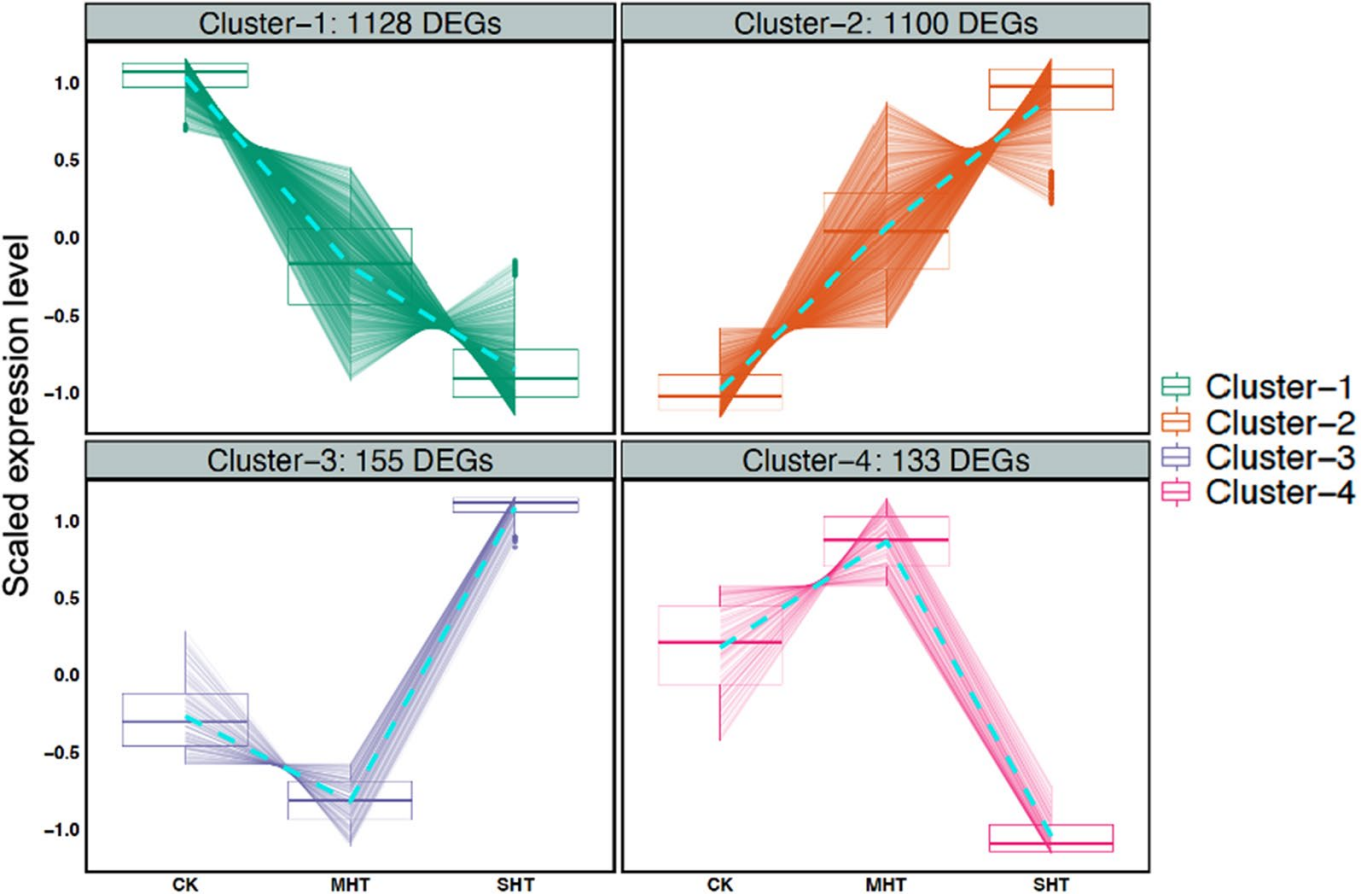
The expression patterns of the DEGs in four clusters across the three treatments. Box plots with distinguishable colors elucidate the distribution of the scaled expression levels of the DEG in CK, MHT, and SHT for each cluster. A dashed line connects the means of the scaled expression levels for each treatment to illustrate the trend of changes. The number of DEGs in each cluster is shown at the top of each plot

To investigate the effects of increasing intensity of HS on whole genome gene expression of *P. tuoliensis*, we divided the 2724 DEGs into four clusters based on their expression patterns in the three groups: CK, MHT, and SHT. Figure 5 illustrates the four clusters and their expression profiles. Cluster-1 contained 1232 DEGs that exhibited a declining trend of expression in both MHT and SHT compared to CK, indicating that these DEGs were suppressed by HS. Cluster-2 consisted of 1191 DEGs that showed an increasing trend of expression in both MHT and SHT compared to CK, suggesting that these DEGs were over-expressed under HS. Cluster-3 included 166 DEGs that exhibited a different response to MHT and SHT compared to CK: they were down-regulated in MHT but were up-regulated in SHT, implying that these DEGs were sensitive to the severity of HS. However, Cluster-4 comprised 135 DEGs that featured an opposite response to MHT and SHT compared to CK, in terms of up-regulation in MHT yet down-regulation in SHT, which may be adaptive to the mild HS but not the severe HS.

### Gene functional enrichment analysis

**Fig. 6:**
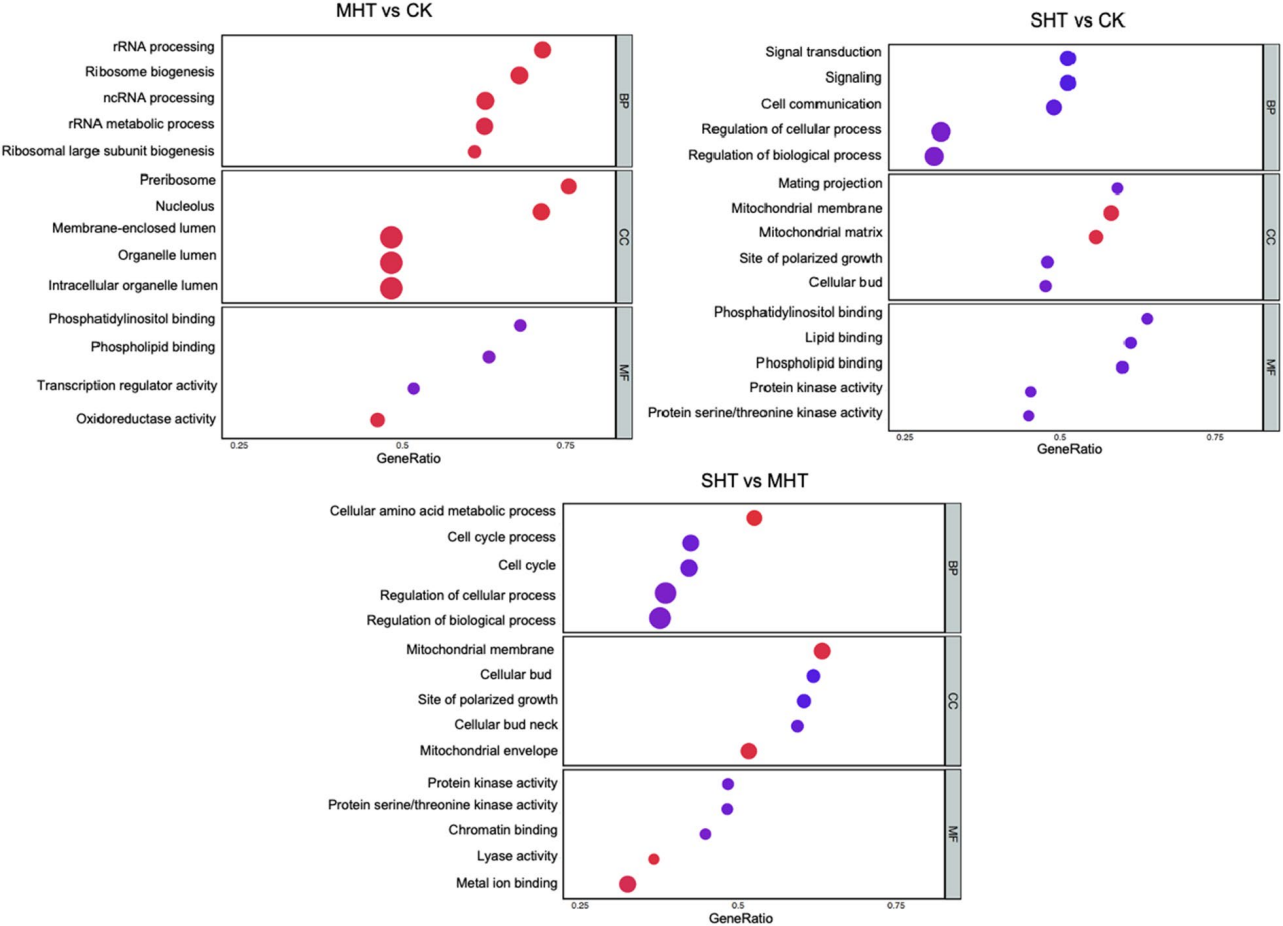
The enrichment of the top 5 GO terms in BP, CC, and MF subontologies for each comparison. The y-axis shows the enriched GO terms, and the x-axis represents the gene ratio of the number of contributing genes to the size of the pre-defined gene set for each GO term. The dot size reflects the number of contributing genes, and the dot color indicates the normalized enrichment score (NES) of each GO term

We performed gene functional enrichment analysis using the *S. cerevisiae* database as references*.* Out of 11,265 unigenes, 1748 were annotated successfully. Following the protocol of GSEA analysis, we analyzed the enrichment of GO and KEGG terms for each pair-wise comparison, and obtained six results of enrichment analyses in total. The GSEA results varied across the comparisons due to the different ranking of gene lists. We revealed 48, 113 and 105 enriched GO terms for MHT&CK, SHT&CK, and SHT&MHT, respectively. Figure 6 depicts the top 5 enriched GO terms (with the lowest adjusted *p*-values) for each comparison in three subontologies, in terms of biological process (BP), cellular component (CC), and molecular function (MF). The interactions between enriched GO terms were illustrated in Additional file 1: Fig. S3 as many pre-defined gene sets have similar contributing genes.

For the comparison of MHT&CK, the top 5 enriched GO terms in BP and CC subontologies had positive normalized enrichment score (NES) values, indicating up-regulation of the core enriched genes. Note, only four GO terms were enriched for MF subontology. Among them, only oxidoreductase activity had a positive NES, while the other three had negative NES values, implying down-regulations of corresponding genes. The average gene ratio of the top enriched GO terms was 0.65 for BP, 0.58 for CC, and 0.57 for MF.

The GSEA of SHT&CK revealed significant down-regulations of genes participating BPs such as signal transduction, cell communication, and regulation-related processes. In addition, the top 5 GO terms in the MF subontology possessed negative NES (Fig. 6). Within CC subontology, only gene sets related to the mitochondrial membrane and matrix had positive NES. The gene ratio of top 5 terms in each subontology was 0.42, 0.54, and 0.55 respectively.

The GSEA between the two HS treatments revealed a total of 106 enriched GO terms, with most of the top enriched terms in each subontology having negative NES. Similar to the findings of SHT&CK analysis, the SHT&MHT comparison also found up-regulation of genes contributing to the mitochondrial membrane gene set. Besides, genes associated with cellular amino acid metabolic process, mitochondrial envelope, lyase activity and metal ion binding were up-regulated. The gene ratios of the top 5 enriched GO terms were relatively lower in the SHT&MHT comparison than the other two comparisons, with an average of 0.43, 0.59, and 0.42 in the BP, CC, and MF subontologies respectively.

**Fig. 7:**
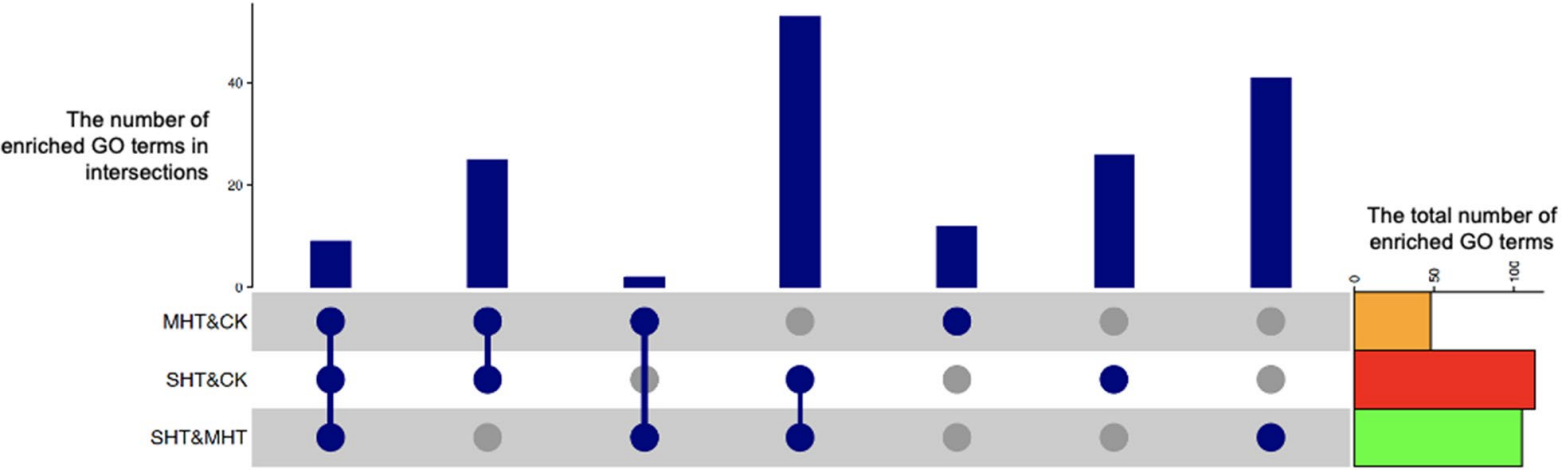
Upset plots of the interactions between GSEA results of three comparisons: MHT&CK, SHT&CK, and SHT&MHT. The bar graph at the top shows the number of GO terms in each intersection at the bottom. The connected blue dots in a column represent a specific intersection between the three comparisons. A single blue dot indicates the enriched GO terms that were only found in the corresponding comparison. The bar graph on the right shows the size of the entire set of enriched GO terms revealed in each comparison

To further investigate the interactions between the enriched GO terms in the three comparisons, we analyzed the overlaps and unique terms among the GSEA results (Fig. 7). A total of 53 GO terms were common to both the SHT&CK and SHT&MHT comparisons, while 25 enriched GO terms were shared between the MHT&CK and SHT&CK comparisons. The MHT&CK and SHT&MHT comparisons had only 2 enriched GO terms in common. In addition, the number of unique enriched GO terms in the GSEA results of MHT&CK, SHT&CK, and SHT&MHT was 12, 26, and 41 respectively. Additionally, all three GSEA results identified 9 common enriched GO terms of which 8 GO terms belonged to the BP subontology and included cell communication, cellular amino acid metabolic process, intracellular signal transduction, regulation of cellular process, signal transduction, signaling, small molecule biosynthetic process, and small molecule metabolic process (Table 2). Note that the GO terms for these two identical GO terms (GO: 0007165 and GO: 0023052) were merged for subsequent interpretation. The only GO term in the CC subontology that was enriched in all three comparisons was mitochondrial matrix.

**Table 2:** Enriched GO terms shared by three comparisons

Subontology	Description	Gene set size	No. of contributing genes			NES^*^		
			MHT&CK	SHT&CK	SHT&MHT	MHT&CK	SHT&CK	SHT&MHT
BP	Cell communication	98	39	48	36	− 1.65	− 2.36	− 2.05
BP	Cellular amino acid metabolic process	78	31	36	41	1.60	1.97	2.22
BP	Intracellular signal transduction	72	28	37	29	− 1.63	− 2.50	− 2.17
BP	Regulation of cellular process	270	81	84	105	− 1.30	− 1.97	− 1.88
BP	Signal transduction	82	33	42	32	− 1.72	− 2.53	− 2.18
BP	Signaling	82	33	42	32	− 1.72	− 2.53	− 2.18
BP	Small molecule biosynthetic process	93	36	49	43	1.62	1.84	1.87
BP	Small molecule metabolic process	175	58	79	88	1.46	1.78	1.88
CC	Mitochondrial matrix	61	25	34	26	1.60	1.87	1.85

*NES normalized enrichment score

The number of genes contributing to the same gene set of the 9 enriched GO terms varied vastly in three comparisons (Table 2). The SHT&CK comparison showed the largest number of contributing genes in 7 of 9 gene sets. In the remaining two gene sets, cellular amino acid metabolic process and regulation of cellular process, the SHT&MHT comparison possessed the largest size of contributing genes, covering 53% and 39% correspondingly. The MHT&CK analysis contained the fewest contributing genes in 6 of 9 gene sets. The NES suggested consistent trends in gene expression alterations within the 9 gene sets. Positive NES indicated up-regulations of genes associated with GO terms such as small molecule biosynthetic process, cellular amino acid metabolic process, and mitochondrial matrix. However, gene sets such as intracellular signal transduction, signal transduction, signaling, regulation of cellular process, and cell communication implied that their contributing genes were down-regulated.

**Fig. 8:**
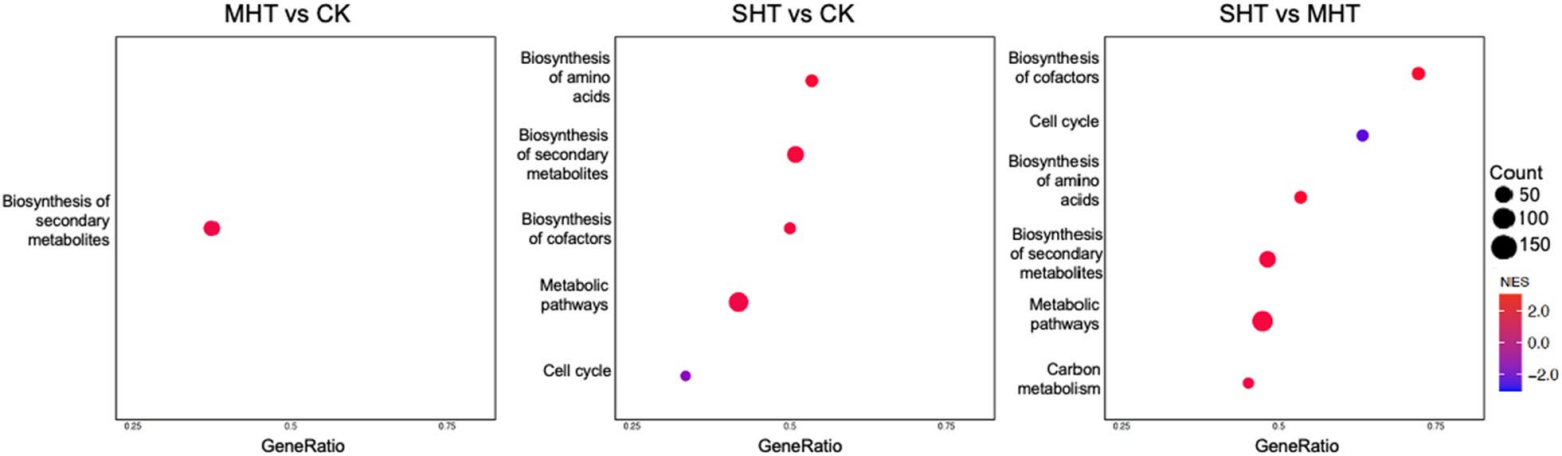
Dot plots showing the enriched pathways discovered in the GSEA results of the MHT&CK, SHT&CK, and SHT&MHT comparisons respectively. The y-axis shows the enriched KEGG pathways, and the x-axis represents the gene ratio of the number of contributing genes to the size of the pre-defined gene set for each KEGG pathway. The dot size reflects the number of contributing genes, and the dot color indicates the normalized enrichment score (NES) of each KEGG pathway

The number of enriched pathways in three comparisons was rather scarce compared to the number of enriched GO terms (Fig. 8). The biosynthesis of secondary metabolites pathway was enriched in all three comparisons with positive NES. It was also the only enriched pathway found in the MHT&CK analysis. The SHT&MHT analysis revealed 6 enriched KEGG pathways, including all 5 pathways enriched in the SHT&CK and one unique pathway: carbon metabolism. The cell cycle pathway showed negative NES in both the SHT&CK and SHT&MHT analyses.

Gene ratios of overlapping enriched pathways varied in each analysis, indicating different numbers of contributing genes detected between comparisons due to alterations in expression levels. For the biosynthesis of secondary metabolites pathway, the SHT&CK comparison had a slightly higher gene ratio of 0.51 than the other two comparisons. The SHT&MHT analysis revealed the highest overall gene ratio of 0.72 among all enriched pathways, including 26 contributing genes for biosynthesis of cofactors. The cell cycle pathway in the SHT&MHT analysis had the second-highest gene ratio of 0.63, almost twice that of the SHT&CK analysis for the same pathway. Similar numbers of contributing genes were identified for biosynthesis of amino acids and metabolic pathways in both SHT&CK and SHT&MHT analyses. The carbon metabolism pathway, only enriched in the SHT&MHT comparison, had 14 contributing genes covering 45% of the gene set.

### DEGs involved in the enriched GO terms/KEGG pathways

We identified the DEGs involved in the enriched GO terms/KEGG pathways, and categorized them to screen for the key genes potentially responsible for HS response. A combined analysis detected common contributing DEGs for each overlapping GO term shared by the three comparisons. In total, 73 DEGs that were categorized in supplementary tables (Additional file 1: Tables S3–S10). The regulation of cellular process had the greatest number of 34 shared contributing DEGs, while the mitochondrial matrix GO term had the fewest with 12.

**Fig. 9:**
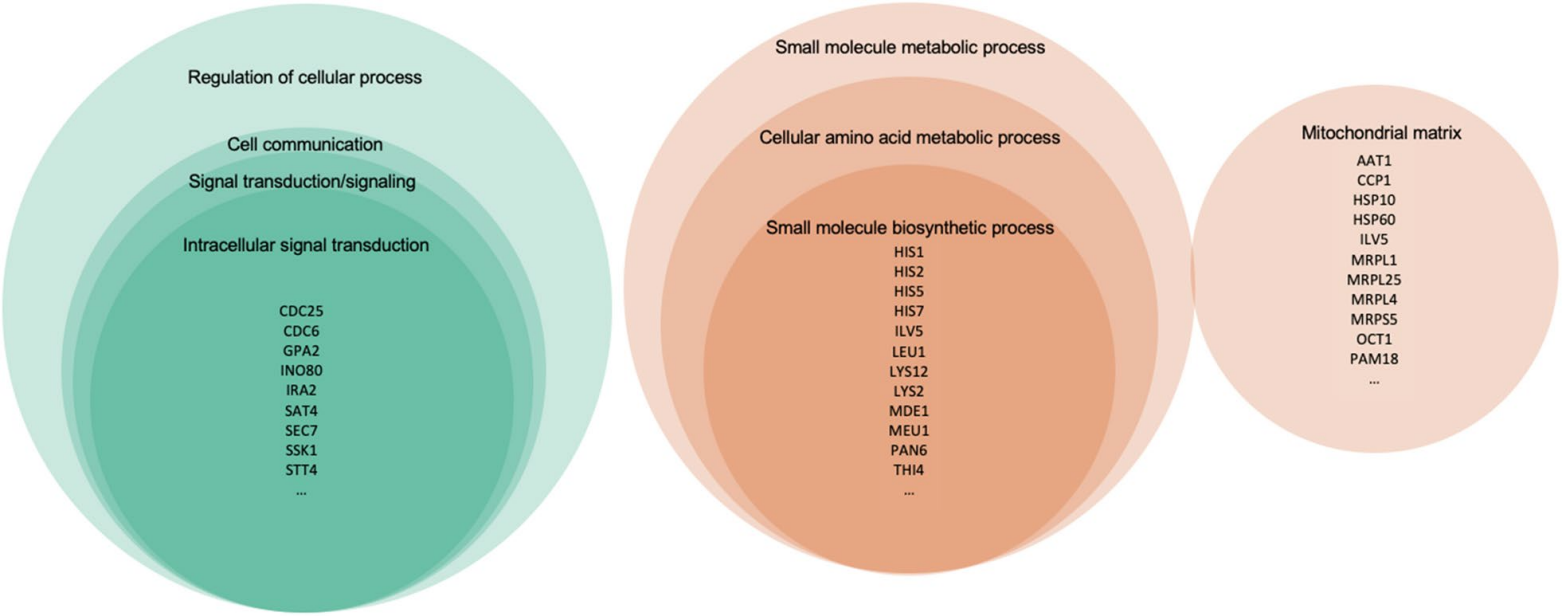
Diagrams of the overlapping DEGs contributing to the 9 enriched GO terms shared by the MHT&CK, SHT&CK, and SHT&MHT comparisons and their intersections. Color represents gene clustering based on the expression patterns: green for Cluster-1 and orange for Cluster-2. The size of intersection areas between circles is proportional to the number of overlapping DEGs. Core genes are listed for the three GO terms with the fewest overlapping contributing DEGs

The number of shared contributing genes was nearly proportional to the size of gene sets. Overlaps of DEGs were noticed in the 9 enriched GO terms (Fig. 9), and were divided into two groups by gene expression patterns. A group of 33 Cluster-1 DEGs contributed to regulation of cellular process, cell communication, signal transduction, signaling, and intracellular signal transduction. The other group was consisted of 40 Cluster-2 DEGs contributing to the rest 4 enriched GO terms. The regulation of cellular process and small molecule metabolic process were the most inclusive, containing all the contributing DEGs of 3 and 2 GO terms in each group. The mitochondrial matrix contributing DEGs were relatively independent, with only two overlapping DEGs with small molecule metabolic process. We found two DEGs with ambiguous classification but the same annotated function (Gene_20200 and Gene_20204) with an ortholog protein product of phosphatidylinositol-4-kinase (Additional file 1: Tables S3–S5, S9). However, gene_20200 was categorized into Cluster-1while gene_20204 was in Cluster-2 based on their expression patterns.

For the overlapping enriched pathway of biosynthesis of secondary metabolites, 31 DEGs were identified in three comparisons in common, including 25 distinct gene functions as multiple DEGs had the same annotated functions (Table 3). Among the shared DEGs, 14 were involved in the biosynthesis of aspartate, glutamate, histidine, lysine, and leucine. Altogether 8 DEGs participated in the ergosterol biosynthesis. Gene_5002 and Gene_5003 shared the same ortholog protein product of adenylate kinase and were responsible for purine metabolism and ATP homeostasis maintenance. Two other DEGs encoded two critical enzymes in the pantothenate biosynthesis pathway, a precursor of coenzyme A. Gene_843 played crucial role in Heme A biosynthesis, while Gene_17492 encoded a key subunit of the glycine cleavage complex involved in tetrahydrofolate biosynthesis and salvage of. The remaining three DEGs had referenced functions in carbon source utilization and energy production, impacting on cell growth and division. All shared DEGs in Table 3 belonged to Cluster-2, showing overexpression under MHT and SHT conditions.

**Table 3:** Summary table of 31 shared DEGs that contribute to the enriched KEGG pathway detected in all three comparisons

Enriched KEGG pathway	Gene ID	Description	Expression patterns
Biosynthesis of secondary metabolites KEGG ID:01110	Gene_4597	Cytosolic aspartate aminotransferase	Cluster-2
	Gene_443	Aconitase	Cluster-2
	Gene_5002	Adenylate kinase	Cluster-2
	Gene_5003	Adenylate kinase	Cluster-2
	Gene_843	Heme A synthase	Cluster-2
	Gene_3555	C-3 sterol dehydrogenase	Cluster-2
	Gene_17310	C-5 sterol desaturase	Cluster-2
	Gene_22349	Delta(24)-sterol C-methyltransferase	Cluster-2
	Gene_23670	UDP-glucose-4-epimerase/mutarotase	Cluster-2
	Gene_17492	T subunit of the mitochondrial glycine decarboxylase complex	Cluster-2
	Gene_4847	NAD(+)-dependent glutamate synthase	Cluster-2
	Gene_4848	NAD(+)-dependent glutamate synthase	Cluster-2
	Gene_8809	ATP phosphoribosyltransferase	Cluster-2
	Gene_14686	Histidinolphosphatase	Cluster-2
	Gene_8226	Histidinol-phosphate aminotransferase	Cluster-2
	Gene_4773	Imidazole glycerol phosphate synthase	Cluster-2
	Gene_8175	HMG-CoA reductase	Cluster-2
	Gene_8176	HMG-CoA reductase	Cluster-2
	Gene_8173	HMG-CoA reductase	Cluster-2
	Gene_8174	HMG-CoA reductase	Cluster-2
	Gene_1367	Subunit of mitochondrial NAD(+)-dependent isocitrate dehydrogenase	Cluster-2
	Gene_1404	Subunit of mitochondrial NAD(+)-dependent isocitrate dehydrogenase	Cluster-2
	Gene_4707	Isopentenyl diphosphate	Cluster-2
	Gene_17859	Acetohydroxyacid reductoisomerase	Cluster-2
	Gene_1046	Isopropylmalate isomerase	Cluster-2
	Gene_1047	Isopropylmalate isomerase	Cluster-2
	Gene_915	Homo-isocitrate dehydrogenase	Cluster-2
	Gene_8162	Alpha aminoadipate reductase	Cluster-2
	Gene_7715	2-dehydropantoate 2-reductase	Cluster-2
	Gene_26510	Pantoate-beta-alanine ligase	Cluster-2
	Gene_6034	Alpha subunit of heterooctameric phosphofructokinase	Cluster-2

## Discussion

Both MHT and SHT significantly altered gene expression in *P. tuoliensis* mycelial cells. Compared to the control group, SHT induced 2371 DEGs, over twice the number of DEGs induced by MHT. As the HS intensity increases, cells have to regulate more genes to cope with the severe stress and to maintain their normal functions and cellular homeostasis. We also performed DEG analysis between SHT and MHT groups, and identified 829 DEGs. These DEGs warrant further investigation as they may provide deeper insights into the key factors causing loss of viability in cells under SHT but not in cells under MHT.

In total, 1380 DEGs overlapped between two or three DEG analyses (MHT&CK, SHT&CK, and SHT&MHT), roughly a half of all DEGs. Additionally, the analysis between SHT and CK revealed over 7 times more unique DEGs compared to the other comparisons (Fig. 4b), indicating a different scale of global gene expression alteration under HS at 36 °C.

The study classified all DEGs into four clusters based on their expression patterns in three treatments. The number of DEGs that were down-regulated in both MHT and SHT groups (Cluster-1) is slightly higher than the number of DEGs that were up-regulated in both HS treatments (Cluster-2) compared to CK. Furthermore, the sum of DEGs in Cluster-1 and Cluster-2 accounts for almost 82% of all DEGs, implying consistent trends in mycelial gene regulations when confronting HS. Cluster-3 contains 155 DEGs that were downregulated under 32 °C but were over-expressed under 36 °C, while Cluster-4 contains 133 DEGs that showed the opposite pattern and were up-regulated and down-regulated in MHT and SHT with respect to CK. Despite the low number of DEGs in Cluster-3 and Cluster-4, these DEGs can be crucial for future studies because they exhibit a distinguishable expression tendency that may be connected to the distinct growth performance observed between MHT and SHT.

We performed DEG analysis and GSEA to identify DEGs that showed functional enrichment in each pair-wise comparison and their interactions between the enrichment results. Out of 2724 DEGs, we found ortholog genes of *S. cerevisiae* for 295 of them. Moreover, we identified 9 overlapping GO terms and 1 overlapping KEGG pathway across three comparisons that provide valuable information into the fungal HS response from the prospective of gene expression. These overlapping enrichment items indicate several biological processes, such as cell communication, cellular amino acid metabolic process, intracellular signal transduction, small molecule biosynthesis, and others, play pivotal roles in transcriptomic response to HS in mycelial cells. Plus, mitochondria is a key organelle during HS response. Based on these findings, we focused on several gene families, gene networks, and their expression patterns that deserve further investigation. Following are some highlights we would like to point out for discussion:

### Variation in expression changes of cell cycle-related DEGs

We identified 7 DEGs that encode ortholog proteins to *S. cerevisiae,* namely Cdc5p, Cdc6p, Cdc14p, and Cdc25p. The expression of the DEG encoding Cdc5p varied in the three treatments, and was assigned to Cluster-4. The other 6 DEGs showed consistent downregulation as the HS intensity increased and follow the Cluster-1 pattern, which were involved in similar enriched GO terms (Table 2). Cdc5p helps cells adapt to the DNA damage checkpoint and suppresses it (Coutelier et al. 2018). Cdc6p is essential for forming and maintaining the pre-replicative complex (pre-RC), which regulates DNA replication initiation and mitotic exit. Likewise, Cdc14p is also indispensable for mitotic exit (Cocker et al. 1996; Taylor et al. 1997; Leatherwood 1998; Stegmeier and Amon 2004). CDC26p participates in Ras protein signal transduction, which is required for mitotic entry and G1 phase progression (Garreau et al. 1996).

Besides the *CDC* family genes, we also found two DEGs that encode the evolutionary conserved Mcm1p, which followed the Cluster-1 trend. They were enriched in regulation of cellular process in all three GSEA analyses (Additional file 1: Table S9). Mcm1p is a transcription regulator that binds to DNA replication origins and regulates G2/M transition (Lydall et al. 1991). Moreover, one DEG was identified encoding Mcm5p that also played a crucial role in DNA replication and cell cycle progression. The expression level of the ortholog *MCM5* elevated in the MHT and SHT, with fold changes of 2.27 and 2.01 compared to CK. Mcm5p is a component of the MCM complex that primes DNA replication origins in G1 and participates in double-strand break repair as well (Aparicio et al. 1997; Bochman and Schwacha 2008).

The identified DEGs associated with cell cycle regulation showed distinct expression patterns under HS conditions. Generally, the genes that facilitate cell cycle progression were down-regulated, while the genes that promote DNA repair and cell cycle restoration were up-regulated (Caspeta et al. 2016). However, the ortholog *Cdc5* showed similar expression level to CK under MHT yet significant down-regulation under SHT. Previous studies have suggested that HS extends the lifespan of budding yeast cells by preventing the accumulation of age-promoting factors in the mother cell, which arrests the progression of normal cell division cycles (Baldi et al. 2017). We hypothesize that severe HS causes irreparable DNA damages in the cells, leading to apoptosis or programmed cell death.

### Overexpression of HSP genes

According to our analysis, *P. tuoliensis* mycelial cells overexpressed two DEGs identical to *S. cerevisiae HSP10* and *HSP60* (both 100% protein sequence identity) under HS conditions at 32 °C and 36 °C. *HSP10* increased 1.74- and 1.93-fold, and *HSP60* increased 3.05- and 3.56-fold under MHT and SHT, respectively, compared to CK. Hsp10 and Hsp60 form a complex that acts as a chaperone and helps the folding of newly synthesized proteins or denatured proteins. This is crucial for maintaining the integrity and stability of mitochondrial proteins and DNA (Tiwari et al. 2015). Thus, the up-regulation of *HSP10* and *HSP60* genes are essential for fungal survival and adaptation to environment changes by enhancing protein folding capacity and preserving mitochondrial function against stress conditions (Caruso Bavisotto et al. 2020).

However, excessive HSPs may also impair fungal growth and development by interfering the normal regulation of cellular processes, such as cell cycle progression, mitochondrial morphology, and respiratory function (Mühlhofer et al. 2019). Our study revealed the mycelium cells had similar viability to the control under MHT, yet their growth was almost halted under SHT. This might be due to the overexpressed HSPs disrupting the normal cellular maintenance. Therefore, the regulation of HSP expression is crucial for maintaining cellular homeostasis.

### Ergosterol biosynthesis and thermotolerance

Our transcriptional analysis identified in total 11 DEGs related to ergosterol biosynthesis with ortholog protein products including Erg3p, Erg4p, Erg5p, Erg6p, Erg26p, Hmg1p and Idi1p. Among them, 9 DEGs were upregulated in both MHT and SHT, while 2 DEGs encoding Erg5p (C-22 sterol desaturase) showed controversial alterations in MHT and SHT. The expression level of evolutionary conserved *ERG5* increased by 1.65 folds in MHT yet reduced by 75% in SHT. Note Erg5p is a key enzyme involved in the last four steps of converting fecosterol to ergosterol. Based on the growth data, we hypothesized that the distinguished expression of *ERG5* may affect ergosterol biosynthesis under HS at 32 °C and 36 °C, which may eventually lead to the varied thermotolerance. Furthermore, in *S. cerevisiae* and *Penicillium oxalicum,* their mutants *erg5* Δ showed significantly faster growth than the wild type at 39.5° and 37°, connecting *Erg5* to the acquisition of persistent thermotolerance (Liu et al. 2017).

Ergosterol levels and composition need to be adjusted when mycelial cells are exposed to HS or other stresses to maintain the membrane fluidity and integrity (Pan et al. 2019). However, the failure to balancing ergosterol turnover may deteriorate fungal membrane permeability, and increase the outflow of intracellular electrolytes causing apoptosis-like cell death (Luo et al. 2021). A previous study has proved that higher ergosterol levels were associated with lifted thermotolerance in yeast due to enhanced membrane rigidity (Swan and Watson 1998). Therefore, further investigations are needed to confirm the substantial ergosterol level in *P. tuoliensis* mycelia under the MHT and SHT conditions.

In conclusion, HS elicits a robust transcriptional response in *P. tuoliensis* mycelial cells by modulating a diversity of genes. The intensity of HS vastly influences gene differential expression. Through the comparative DEGs analysis combined with gene function enrichment analysis under MHT and SHT conditions, we revealed critical biological processes and pathways potentially related to viability maintenance and thermotolerance acquisition for mycelial cells against HS. In addition to previous studies on mushroom response to HS, we found genes involved in cell cycle regulation and ergosterol biosynthesis may also play vital roles in the HS response process, which are worthy for further explorations to elucidate the molecular mechanisms of HS response in mushrooms.

### Supplementary Information

**Additional file 1**: Supplemetary materials.

## Data Availability

The authors confirm that all the experimental data are available and accessible via the main text and/or the supplementary information.
