# Supplementary material for: Comparative analysis of genome-wide transcriptional responses to continuous heat stress in Pleurotus tuoliensis
Source: AMB Express. 2023 Nov 2;13:121. doi: 10.1186/s13568-023-01630-y (PMC10622393; doi:10.1186/s13568-023-01630-y)
Supplement: Supplementary file 1 — Additional file 1: Supplemetary materials. [file 13568_2023_1630_MOESM1_ESM.docx]

Submitted to: **AMB Express**

**Comparative Analysis of Genome-wide Transcriptional Responses to Continuous Heat Stress in *Pleurotus tuoliensis***

Long Chen^a,b,*^, Ying Luo^a^, Jiazheng Li^a,b^, Zhijun Zhang^a,b^, Di Wu^c,*^

*^a^Tianjin Academy of Agricultural Sciences, Tianjin 300192, China;*

*^b^National Engineering Technology Research Center for Preservation of Agricultural Products, Tianjin 300384, China;*

*^c^Bionano Genomics, San Diego, CA 92121, USA*

Email addresses: [lchen6316@gmail.com](mailto:lchen6316@gmail.com) (L. Chen), [luoying201@163.com](mailto:luoying201@163.com) (Y. Luo), [lijzh163@163.com](mailto:lijzh163@163.com) (J. Li), [tjzhangzj@sina.cn](mailto:tjzhangzj@sina.cn) (Z. Zhang), [dwu@bionano.com](mailto:dwu@bionano.com) (D. Wu).

* Correspondence: [*lchen6316@gmail.com*](mailto:lchen6316@gmail.com), *+86 159-2215-1902 (L. Chen)*; [*dwu@bionano.com*](mailto:dwu@bionano.com), +86 13388011318*(D. Wu)*


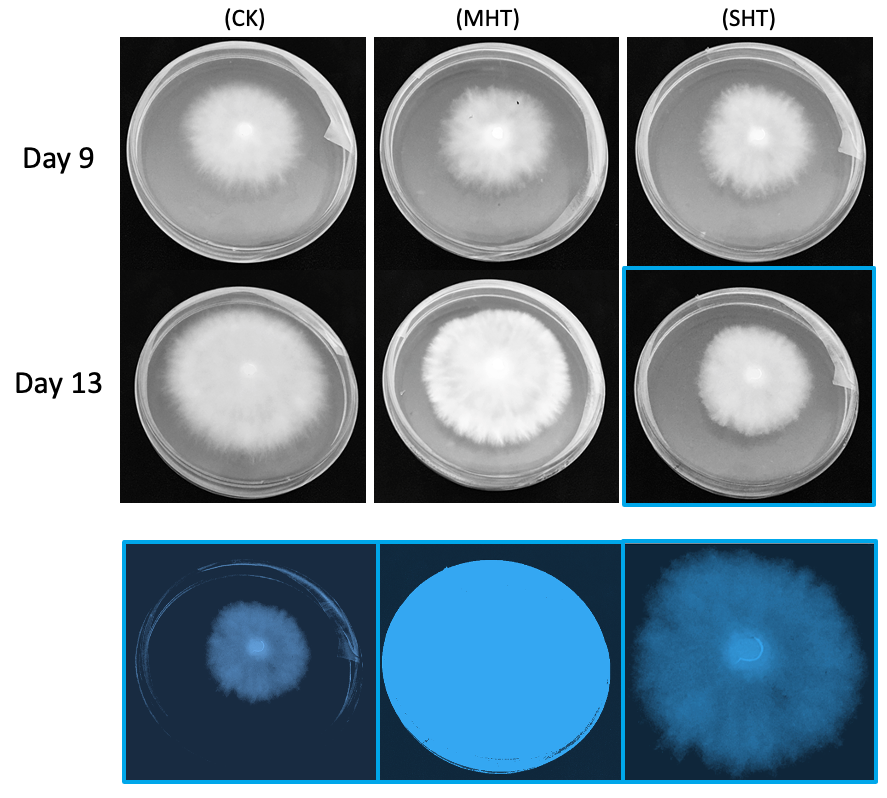


**Fig. S1** Images of one replicate in each group on the Day 9 (before HS) and Day 13 (after 96 h HS). The bottom is an illustration of the SHT plate on Day 13 (marked with blue border) showing the captured pixels of plate, dish area, and colony area separately using the NCAR algorithm.

**Table S1** Summary statistics of rRNA alignments of sequencing samples

| **Sample** | **Total paired reads** | **Paired mapped reads** | **Unpaired mapped reads** | **Unmapped reads** | **Total mapped** |
| --- | --- | --- | --- | --- | --- |
| CK1 | 16904798 | 452574(2.68%) | 3940(0.02%) | 16448284(97.30%) | 456514(2.70%) |
| CK2 | 22009157 | 800154(3.64%) | 4326(0.02%) | 21204676(96.34%) | 804480(3.66%) |
| CK3 | 21485183 | 765586(3.56%) | 6772(0.03%) | 20712826(96.41%) | 772358(3.59%) |
| MHT1 | 26032345 | 914316(3.51%) | 4846(0.02%) | 25113184(96.47%) | 919162(3.53%) |
| MHT2 | 23893148 | 753816(3.15%) | 3980(0.02%) | 23135352(96.83%) | 757796(3.17%) |
| MHT3 | 23676604 | 740331(3.13%) | 4684(0.02%) | 22931588(96.85%) | 745016(3.15%) |
| SHT1 | 26241571 | 965670(3.68%) | 8378(0.03%) | 25267523(96.29%) | 974048(3.71%) |
| SHT2 | 23261438 | 666341(2.86%) | 5490(0.02%) | 22589607(97.11%) | 671831(2.89%) |
| SHT3 | 23375530 | 1136569(4.86%) | 8999(0.04%) | 22229962(95.10%) | 1145568(4.90%) |

**Table S2** Summary statistics of reads alignments to the reference genome (Pleurotus tuoliensis strain JKBL130LB)

| **Sample** | **Total paired reads** | **Paired mapped reads** | **Unpaired mapped reads** | **Unmapped reads** | **Total mapped** |
| --- | --- | --- | --- | --- | --- |
| CK1 | 16445087 | 14447587(87.85%) | 472820(2.88%) | 1524680(9.27%) | 14920406(90.73%) |
| CK2 | 21201213 | 18750858(88.44%) | 587616(2.77%) | 1862738(8.79%) | 19338474(91.21%) |
| CK3 | 20707331 | 18173756(87.76%) | 504773(2.44%) | 2028802(9.80%) | 18678529(90.20%) |
| MHT1 | 25109109 | 22368127(89.08%) | 639400(2.55%) | 2101582(8.37%) | 23007528(91.63%) |
| MHT2 | 23131998 | 20642175(89.24%) | 569518(2.46%) | 1920305(8.30%) | 21211693(91.70%) |
| MHT3 | 22927660 | 20409694(89.02%) | 566607(2.47%) | 1951359(8.51%) | 20976301(91.49%) |
| SHT1 | 25260431 | 22552223(89.28%) | 610835(2.42%) | 2097373(8.30%) | 23163058(91.70%) |
| SHT2 | 22584926 | 20063695(88.84%) | 541668(2.40%) | 1979564(8.76%) | 20605362(91.24%) |
| SHT3 | 22222415 | 19848574(89.32%) | 563512(2.54%) | 1810330(8.15%) | 20412086(91.85%) |


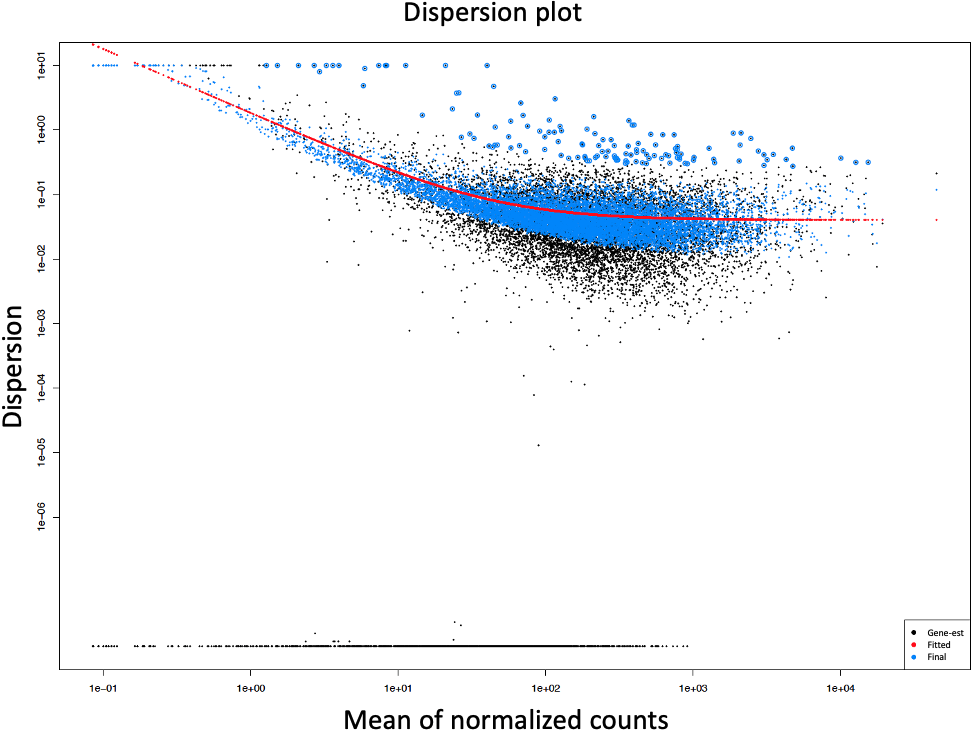


**Fig. S2** A dispersion plot of means of normalized counts. Each gene is represented by a black dot, showing its dispersion estimate as a function of its mean normalized count. A curve is fitted to the gene-wise dispersion estimates, indicating the expected dispersion value for genes with a given normalized count. The gene-wise dispersion estimates are then shrunk by an empirical Bayes approach towards the fitted curve, resulting in the final dispersion estimates shown by the blue dots. The genes that have higher variability than expected are not shrunk towards the curve. These outliers are marked by blue circles in the dispersion plot.


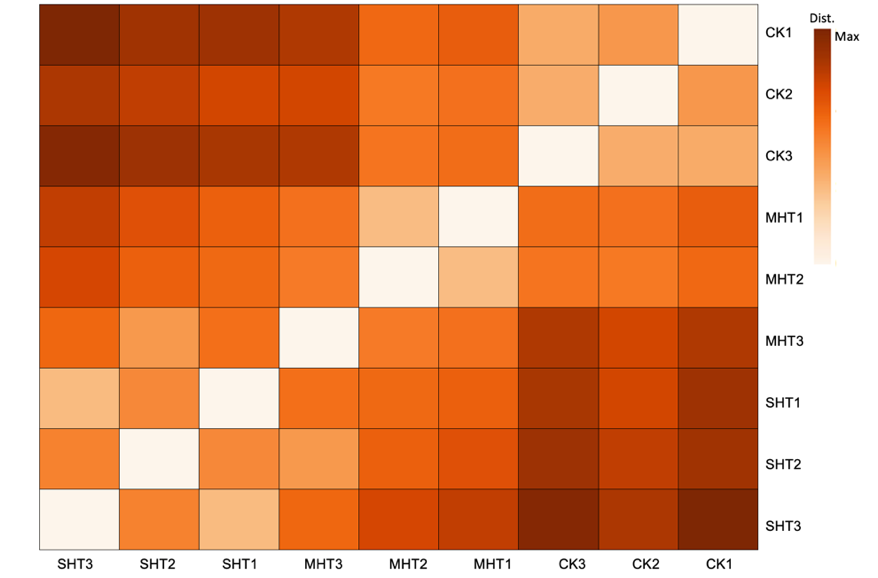


**Fig. S3** A heatmap depicts orthogonal distances between nine samples based on the normalized counts of the entire 11,265 unigenes. The darkest color indicates the maximum distance between two individual samples.


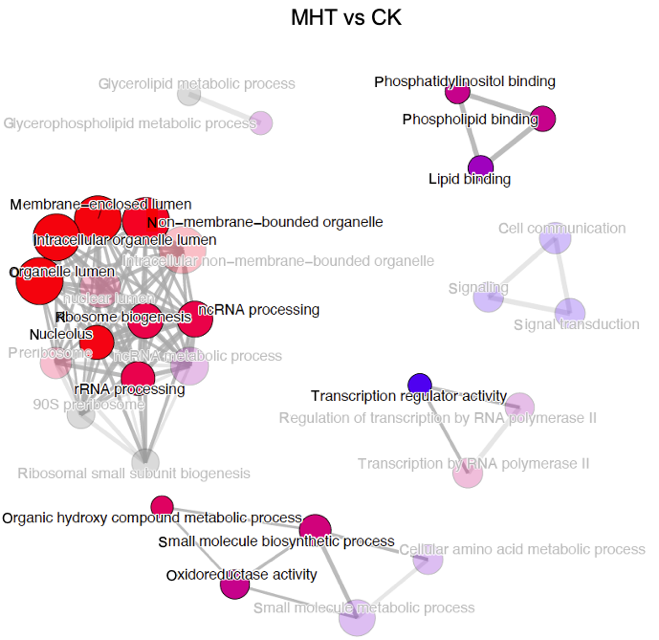

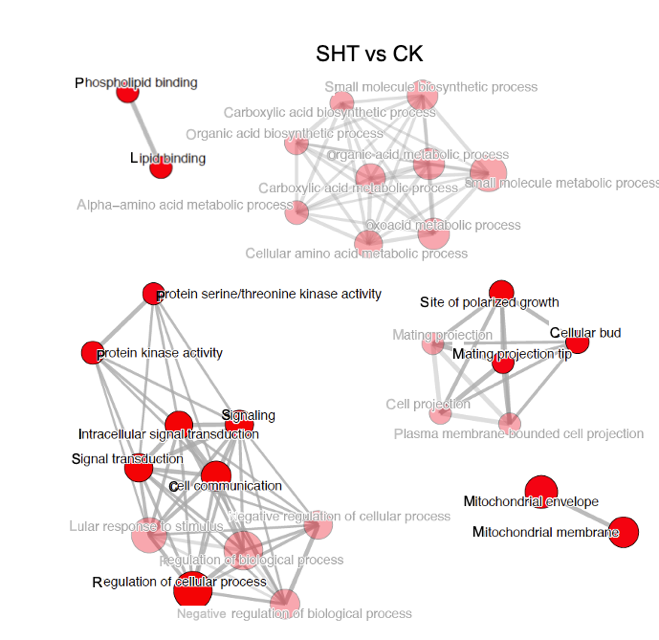

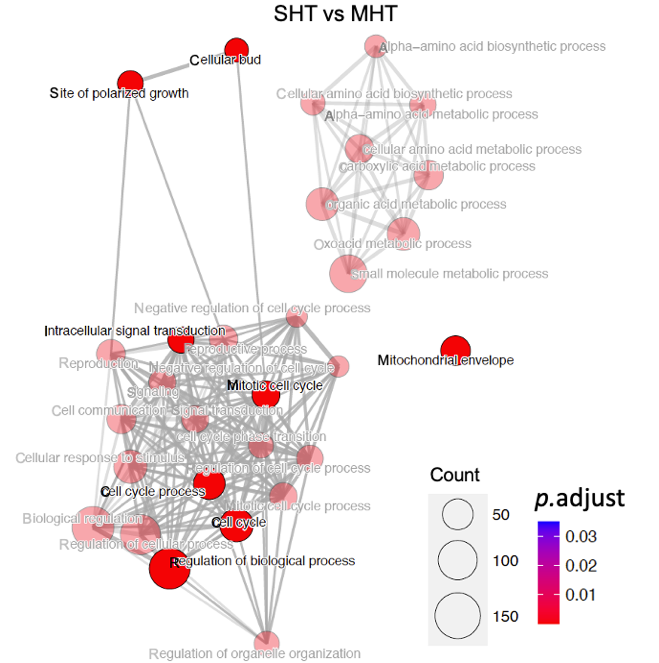


**Fig. S4** Enrichment maps of the top 30 GO terms. Each node represents an enriched gene set with its size proportional to the number of the detected genes in that set. Grey lines connecting nodes indicate similarities between the two gene sets: the thicker and shorter the line, the more similar the gene sets. Node color stands for the adjust *p*-value. Highlighted nodes indicate the top 5 GO terms in each category of BP, CC, and MF shown in Figure 6.

**Table S3** Summary table of the DEGs belong to the enriched GO term of oxidoreductase activity.

| **Enriched GO term** | **Gene ID** | **Description** | **Expression patterns** |
| --- | --- | --- | --- |
| **Cell communication**  **GO: 0007154** | Gene_25128 | Membrane bound guanine nucleotide exchange factor | Cluster-1 |
|  | Gene_25129 | Membrane bound guanine nucleotide exchange factor | Cluster-1 |
|  | Gene_7304 | Essential ATP-binding protein required for DNA replication | Cluster-1 |
|  | Gene_7303 | Essential ATP-binding protein required for DNA replication | Cluster-1 |
|  | Gene_6973 | Transcriptional regulator | Cluster-1 |
|  | Gene_6972 | Transcriptional regulator | Cluster-1 |
|  | Gene_11731 | Nucleotide binding alpha subunit of the heterotrimeric G protein | Cluster-1 |
|  | Gene_4499 | Nucleosome spacing factor | Cluster-1 |
|  | Gene_1628 | GTPase-activating protein | Cluster-1 |
|  | Gene_25709 | Ser/Thr protein kinase involved in salt tolerance, nutrient response | Cluster-1 |
|  | Gene_25710 | Ser/Thr protein kinase involved in salt tolerance, nutrient response | Cluster-1 |
|  | Gene_781 | Guanine nucleotide exchange factor (GEF) for ADP ribosylation factors | Cluster-1 |
|  | Gene_783 | Guanine nucleotide exchange factor (GEF) for ADP ribosylation factors | Cluster-1 |
|  | Gene_784 | Guanine nucleotide exchange factor (GEF) for ADP ribosylation factors | Cluster-1 |
|  | Gene_785 | Guanine nucleotide exchange factor (GEF) for ADP ribosylation factors | Cluster-1 |
|  | Gene_4896 | Key component of the RAM signaling network | Cluster-1 |
|  | Gene_4897 | Key component of the RAM signaling network | Cluster-1 |
|  | Gene_4898 | Key component of the RAM signaling network | Cluster-1 |
|  | Gene_1194 | Cytoplasmic phosphorelay intermediate osmosensor and regulator | Cluster-1 |
|  | Gene_1195 | Cytoplasmic phosphorelay intermediate osmosensor and regulator | Cluster-1 |
|  | Gene_20200 | Phosphatidylinositol-4-kinase | Cluster-1 |
|  | Gene_20204 | Phosphatidylinositol-4-kinase | Cluster-2 |

**Table S4** Summary table of the DEGs that contribute to the enriched GO term of intracellular signal transduction.

| **Enriched GO term** | **Gene ID** | **Description** | **Expression patterns** |
| --- | --- | --- | --- |
| **Intracellular signal transduction**  **GO: 0035556** | Gene_25128 | Membrane bound guanine nucleotide exchange factor | Cluster-1 |
|  | Gene_25129 | Membrane bound guanine nucleotide exchange factor | Cluster-1 |
|  | Gene_7304 | Essential ATP-binding protein required for DNA replication | Cluster-1 |
|  | Gene_7303 | Essential ATP-binding protein required for DNA replication | Cluster-1 |
|  | Gene_11731 | Nucleotide binding alpha subunit of the heterotrimeric G protein | Cluster-1 |
|  | Gene_4499 | Nucleosome spacing factor | Cluster-1 |
|  | Gene_1628 | GTPase-activating protein | Cluster-1 |
|  | Gene_25709 | Ser/Thr protein kinase involved in salt tolerance, nutrient response | Cluster-1 |
|  | Gene_25710 | Ser/Thr protein kinase involved in salt tolerance, nutrient response | Cluster-1 |
|  | Gene_781 | Guanine nucleotide exchange factor (GEF) for ADP ribosylation factors | Cluster-1 |
|  | Gene_783 | Guanine nucleotide exchange factor (GEF) for ADP ribosylation factors | Cluster-1 |
|  | Gene_784 | Guanine nucleotide exchange factor (GEF) for ADP ribosylation factors | Cluster-1 |
|  | Gene_785 | Guanine nucleotide exchange factor (GEF) for ADP ribosylation factors | Cluster-1 |
|  | Gene_1194 | Cytoplasmic phosphorelay intermediate osmosensor and regulator | Cluster-1 |
|  | Gene_1195 | Cytoplasmic phosphorelay intermediate osmosensor and regulator | Cluster-1 |
|  | Gene_20200 | Phosphatidylinositol-4-kinase | Cluster-1 |
|  | Gene_20204 | Phosphatidylinositol-4-kinase | Cluster-2 |

**Table S5** Summary table of the DEGs that contribute to the enriched GO terms of signaling and signal transduction.

| **Enriched GO term** | **Gene ID** | **Description** | **Expression patterns** |
| --- | --- | --- | --- |
| **Signaling**  **GO:0023052**  **Signal transduction**  **GO:0007165** | Gene_7304 | Essential ATP-binding protein required for DNA replication | Cluster-1 |
|  | Gene_7303 | Essential ATP-binding protein required for DNA replication | Cluster-1 |
|  | Gene_25128 | Membrane bound guanine nucleotide exchange factor | Cluster-1 |
|  | Gene_25129 | Membrane bound guanine nucleotide exchange factor | Cluster-1 |
|  | Gene_11731 | Nucleotide binding alpha subunit of the heterotrimeric G protein | Cluster-1 |
|  | Gene_4499 | Nucleosome spacing factor | Cluster-1 |
|  | Gene_1628 | GTPase-activating protein | Cluster-1 |
|  | Gene_25709 | Ser/Thr protein kinase involved in salt tolerance, nutrient response | Cluster-1 |
|  | Gene_25710 | Ser/Thr protein kinase involved in salt tolerance, nutrient response | Cluster-1 |
|  | Gene_781 | Guanine nucleotide exchange factor (GEF) for ADP ribosylation factors | Cluster-1 |
|  | Gene_783 | Guanine nucleotide exchange factor (GEF) for ADP ribosylation factors | Cluster-1 |
|  | Gene_784 | Guanine nucleotide exchange factor (GEF) for ADP ribosylation factors | Cluster-1 |
|  | Gene_785 | Guanine nucleotide exchange factor (GEF) for ADP ribosylation factors | Cluster-1 |
|  | Gene_4896 | Key component of the RAM signaling network | Cluster-1 |
|  | Gene_4897 | Key component of the RAM signaling network | Cluster-1 |
|  | Gene_4898 | Key component of the RAM signaling network | Cluster-1 |
|  | Gene_1194 | Cytoplasmic phosphorelay intermediate osmosensor and regulator | Cluster-1 |
|  | Gene_1195 | Cytoplasmic phosphorelay intermediate osmosensor and regulator | Cluster-1 |
|  | Gene_20200 | Phosphatidylinositol-4-kinase | Cluster-1 |
|  | Gene_20204 | Phosphatidylinositol-4-kinase | Cluster-2 |

**Table S6** Summary table of the DEGs that contribute to the enriched GO term of small molecule biosynthetic process.

| **Enriched GO term** | **Gene ID** | **Description** | **Expression patterns** |
| --- | --- | --- | --- |
| **Small molecule biosynthetic process**  **GO: 0044283** | Gene_8809 | ATP phosphoribosyltransferase | Cluster-2 |
|  | Gene_14686 | Histidinolphosphatase | Cluster-2 |
|  | Gene_8226 | Histidinol-phosphate aminotransferase | Cluster-2 |
|  | Gene_4773 | Imidazole glycerol phosphate synthase | Cluster-2 |
|  | Gene_17859 | Acetohydroxyacid reductoisomerase and mtDNA binding protein | Cluster-2 |
|  | Gene_1046 | Isopropylmalate isomerase | Cluster-2 |
|  | Gene_1047 | Isopropylmalate isomerase | Cluster-2 |
|  | Gene_915 | Homo-isocitrate dehydrogenase | Cluster-2 |
|  | Gene_8162 | Alpha aminoadipate reductase | Cluster-2 |
|  | Gene_3738 | 5'-methylthioribulose-1-phosphate dehydratase | Cluster-2 |
|  | Gene_331 | Methylthioadenosine phosphorylase (MTAP) | Cluster-2 |
|  | Gene_330 | Methylthioadenosine phosphorylase (MTAP) | Cluster-2 |
|  | Gene_332 | Methylthioadenosine phosphorylase (MTAP) | Cluster-2 |
|  | Gene_26510 | Pantothenate synthase | Cluster-2 |
|  | Gene_3689 | Thiazole synthase | Cluster-2 |

**Table S7** Summary table of the DEGs that contribute to the enriched GO term of small molecule metabolic process.

| **Enriched GO term** | **Gene ID** | **Description** | **Expression patterns** |
| --- | --- | --- | --- |
| **Small molecule metabolic process**  **GO: 0044281** | Gene_4597 | Cytosolic aspartate aminotransferase | Cluster-2 |
|  | Gene_5002 | Adenylate kinase, required for purine metabolism | Cluster-2 |
|  | Gene_5003 | Adenylate kinase, required for purine metabolism | Cluster-2 |
|  | Gene_19876 | Kynurenine 3-monooxygenase | Cluster-2 |
|  | Gene_4600 | Aspartyl-tRNA synthetase, primarily cytoplasmic | Cluster-2 |
|  | Gene_4599 | Aspartyl-tRNA synthetase, primarily cytoplasmic | Cluster-2 |
|  | Gene_17492 | T subunit of the mitochondrial glycine decarboxylase complex | Cluster-2 |
|  | Gene_16967 | GMP synthase | Cluster-2 |
|  | Gene_16966 | GMP synthase | Cluster-2 |
|  | Gene_26362 | Subunit of the trimeric GatFAB AmidoTransferase(AdT) complex | Cluster-2 |
|  | Gene_26363 | Subunit of the trimeric GatFAB AmidoTransferase(AdT) complex | Cluster-2 |
|  | Gene_8809 | ATP phosphoribosyltransferase | Cluster-2 |
|  | Gene_14686 | Histidinolphosphatase | Cluster-2 |
|  | Gene_8226 | Histidinol-phosphate aminotransferase | Cluster-2 |
|  | Gene_4773 | Imidazole glycerol phosphate synthase | Cluster-2 |
|  | Gene_17859 | Acetohydroxyacid reductoisomerase and mtDNA binding protein | Cluster-2 |
|  | Gene_16058 | Lysyl-tRNA synthetase | Cluster-2 |
|  | Gene_1046 | Isopropylmalate isomerase | Cluster-2 |
|  | Gene_1047 | Isopropylmalate isomerase | Cluster-2 |
|  | Gene_915 | Homo-isocitrate dehydrogenase | Cluster-2 |
|  | Gene_8162 | Alpha aminoadipate reductase | Cluster-2 |
|  | Gene_3738 | 5'-methylthioribulose-1-phosphate dehydratase | Cluster-2 |
|  | Gene_331 | Methylthioadenosine phosphorylase (MTAP) | Cluster-2 |
|  | Gene_330 | Methylthioadenosine phosphorylase (MTAP) | Cluster-2 |
|  | Gene_332 | Methylthioadenosine phosphorylase (MTAP) | Cluster-2 |
|  | Gene_7715 | 2-dehydropantoate 2-reductase | Cluster-2 |
|  | Gene_26510 | Pantothenate synthase | Cluster-2 |
|  | Gene_6034 | Alpha subunit of heterooctameric phosphofructokinase | Cluster-2 |
|  | Gene_3689 | Thiazole synthase | Cluster-2 |
|  | Gene_4597 | Cytosolic aspartate aminotransferase | Cluster-2 |
|  | Gene_5002 | Adenylate kinase, required for purine metabolism | Cluster-2 |
|  | Gene_5003 | Adenylate kinase, required for purine metabolism | Cluster-2 |
|  | Gene_19876 | Kynurenine 3-monooxygenase | Cluster-2 |
|  | Gene_4600 | Aspartyl-tRNA synthetase, primarily cytoplasmic | Cluster-2 |
|  | Gene_4599 | Aspartyl-tRNA synthetase, primarily cytoplasmic | Cluster-2 |
|  | Gene_17492 | T subunit of the mitochondrial glycine decarboxylase complex | Cluster-2 |

**Table S8** Summary table of the DEGs that contribute to the enriched GO term of cellular amino acid metabolic process.

| **Enriched GO term** | **Gene ID** | **Description** | **Expression patterns** |
| --- | --- | --- | --- |
| **Cellular amino acid metabolic process**  **GO: 0006520** | Gene_4597 | Cytosolic aspartate aminotransferase | Cluster-2 |
|  | Gene_4600 | Aspartyl-tRNA synthetase, primarily cytoplasmic | Cluster-2 |
|  | Gene_4599 | Aspartyl-tRNA synthetase, primarily cytoplasmic | Cluster-2 |
|  | Gene_17492 | T subunit of the mitochondrial glycine decarboxylase complex | Cluster-2 |
|  | Gene_16967 | GMP synthase | Cluster-2 |
|  | Gene_16966 | GMP synthase | Cluster-2 |
|  | Gene_26362 | Subunit of the trimeric GatFAB AmidoTransferase(AdT) complex | Cluster-2 |
|  | Gene_26363 | Subunit of the trimeric GatFAB AmidoTransferase(AdT) complex | Cluster-2 |
|  | Gene_8809 | ATP phosphoribosyltransferase | Cluster-2 |
|  | Gene_14686 | Histidinolphosphatase | Cluster-2 |
|  | Gene_8226 | Histidinol-phosphate aminotransferase | Cluster-2 |
|  | Gene_4773 | Imidazole glycerol phosphate synthase | Cluster-2 |
|  | Gene_17859 | Acetohydroxyacid reductoisomerase and mtDNA binding protein | Cluster-2 |
|  | Gene_16058 | Lysyl-tRNA synthetase | Cluster-2 |
|  | Gene_1046 | Isopropylmalate isomerase | Cluster-2 |
|  | Gene_1047 | Isopropylmalate isomerase | Cluster-2 |
|  | Gene_915 | Homo-isocitrate dehydrogenase | Cluster-2 |
|  | Gene_8162 | Alpha aminoadipate reductase | Cluster-2 |
|  | Gene_3738 | 5'-methylthioribulose-1-phosphate dehydratase | Cluster-2 |
|  | Gene_331 | Methylthioadenosine phosphorylase (MTAP) | Cluster-2 |
|  | Gene_330 | Methylthioadenosine phosphorylase (MTAP) | Cluster-2 |
|  | Gene_332 | Methylthioadenosine phosphorylase (MTAP) | Cluster-2 |

**Table S9** Summary table of the DEGs that contribute to the enriched GO term of regulation of cellular process.

| **Enriched GO term** | **Gene ID** | **Description** | **Expression patterns** |
| --- | --- | --- | --- |
| **Regulation of cellular process**  **GO: 0050794** | Gene_4499 | Nucleosome spacing factor | Cluster-1 |
|  | Gene_3922 | Transcription factor | Cluster-1 |
|  | Gene_3921 | Transcription factor | Cluster-1 |
|  | Gene_7304 | Essential ATP-binding protein required for DNA replication | Cluster-1 |
|  | Gene_7303 | Essential ATP-binding protein required for DNA replication | Cluster-1 |
|  | Gene_25128 | Membrane bound guanine nucleotide exchange factor | Cluster-1 |
|  | Gene_25129 | Membrane bound guanine nucleotide exchange factor | Cluster-1 |
|  | Gene_781 | Guanine nucleotide exchange factor (GEF) for ADP ribosylation factors | Cluster-1 |
|  | Gene_783 | Guanine nucleotide exchange factor (GEF) for ADP ribosylation factors | Cluster-1 |
|  | Gene_784 | Guanine nucleotide exchange factor (GEF) for ADP ribosylation factors | Cluster-1 |
|  | Gene_785 | Guanine nucleotide exchange factor (GEF) for ADP ribosylation factors | Cluster-1 |
|  | Gene_1194 | Cytoplasmic phosphorelay intermediate osmosensor and regulator | Cluster-1 |
|  | Gene_1195 | Cytoplasmic phosphorelay intermediate osmosensor and regulator | Cluster-1 |
|  | Gene_1655 | Protein phosphatase required for mitotic exit | Cluster-1 |
|  | Gene_4698 | Catalytic subunit of Dcp1p-Dcp2p decapping enzyme complex | Cluster-1 |
|  | Gene_4699 | Catalytic subunit of Dcp1p-Dcp2p decapping enzyme complex | Cluster-1 |
|  | Gene_828 | Subunit of the THO complex | Cluster-1 |
|  | Gene_827 | Subunit of the THO complex | Cluster-1 |
|  | Gene_20200 | Phosphatidylinositol-4-kinase | Cluster-1 |
|  | Gene_20204 | Phosphatidylinositol-4-kinase | Cluster-2 |
|  | Gene_6973 | Transcriptional regulator | Cluster-1 |
|  | Gene_6972 | Transcriptional regulator | Cluster-1 |
|  | Gene_11731 | Nucleotide binding alpha subunit of the heterotrimeric G protein | Cluster-1 |
|  | Gene_1628 | GTPase-activating protein | Cluster-1 |
|  | Gene_25709 | Ser/Thr protein kinase involved in salt tolerance, nutrient response | Cluster-1 |
|  | Gene_25710 | Ser/Thr protein kinase involved in salt tolerance, nutrient response | Cluster-1 |
|  | Gene_4896 | Key component of the RAM signaling network | Cluster-1 |
|  | Gene_4897 | Key component of the RAM signaling network | Cluster-1 |
|  | Gene_4898 | Key component of the RAM signaling network | Cluster-1 |
|  | Gene_26374 | Ubiquitin hydrolase | Cluster-1 |
|  | Gene_26375 | Ubiquitin hydrolase | Cluster-1 |
|  | Gene_19938 | Subunit of the SAGA transcriptional regulatory complex; | Cluster-1 |
|  | Gene_19939 | Subunit of the SAGA transcriptional regulatory complex; | Cluster-1 |

**Table S10** Summary table of the DEGs that contribute to the enriched GO term of mitochondrial matrix.

| **Enriched GO term** | **Gene ID** | **Description** | **Expression patterns** |
| --- | --- | --- | --- |
| **Mitochondrial matrix**  **GO:0044283** | Gene_4597 | Cytosolic aspartate aminotransferase | Cluster-2 |
|  | Gene_7345 | Mitochondrial cytochrome-c peroxidase | Cluster-2 |
|  | Gene_17118 | Mitochondrial matrix co-chaperonin | Cluster-2 |
|  | Gene_19698 | Tetradecameric mitochondrial chaperonin | Cluster-2 |
|  | Gene_17859 | Acetohydroxyacid reductoisomerase and mtDNA binding protein | Cluster-2 |
|  | Gene_23494 | Mitochondrial ribosomal protein of the large subunit | Cluster-2 |
|  | Gene_22257 | Mitochondrial ribosomal protein of the large subunit | Cluster-2 |
|  | Gene_3658 | Mitochondrial ribosomal protein of the large subunit | Cluster-2 |
|  | Gene_22018 | Mitochondrial ribosomal protein of the small subunit | Cluster-2 |
|  | Gene_7687 | Mitochondrial intermediate peptidase | Cluster-2 |
|  | Gene_3172 | Subunit of the import motor (PAM complex) | Cluster-2 |
|  | Gene_18208 | Subunit of the import motor (PAM complex) | Cluster-2 |
